# Identification of novel DNA repair proteins via primary sequence, secondary structure, and homology

**DOI:** 10.1186/1471-2105-10-25

**Published:** 2009-01-20

**Authors:** JB Brown, Tatsuya Akutsu

**Affiliations:** 1 Bioinformatics Center, Institute for Chemical Research, Kyoto University, Uji, Kyoto, 611-0011, Japan

## Abstract

**Background:**

DNA repair is the general term for the collection of critical mechanisms which repair many forms of DNA damage such as methylation or ionizing radiation. DNA repair has mainly been studied in experimental and clinical situations, and relatively few information-based approaches to new extracting DNA repair knowledge exist. As a first step, automatic detection of DNA repair proteins in genomes via informatics techniques is desirable; however, there are many forms of DNA repair and it is not a straightforward process to identify and classify repair proteins with a single optimal method. We perform a study of the ability of homology and machine learning-based methods to identify and classify DNA repair proteins, as well as scan vertebrate genomes for the presence of novel repair proteins. Combinations of primary sequence polypeptide frequency, secondary structure, and homology information are used as feature information for input to a Support Vector Machine (SVM).

**Results:**

We identify that SVM techniques are capable of identifying portions of DNA repair protein datasets without admitting false positives; at low levels of false positive tolerance, homology can also identify and classify proteins with good performance. Secondary structure information provides improved performance compared to using primary structure alone. Furthermore, we observe that machine learning methods incorporating homology information perform best when data is filtered by some clustering technique. Analysis by applying these methodologies to the scanning of multiple vertebrate genomes confirms a positive correlation between the size of a genome and the number of DNA repair protein transcripts it is likely to contain, and simultaneously suggests that all organisms have a non-zero minimum number of repair genes. In addition, the scan result clusters several organisms' repair abilities in an evolutionarily consistent fashion. Analysis also identifies several functionally unconfirmed proteins that are highly likely to be involved in the repair process. A new web service, INTREPED, has been made available for the immediate search and annotation of DNA repair proteins in newly sequenced genomes.

**Conclusion:**

Despite complexity due to a multitude of repair pathways, combinations of sequence, structure, and homology with Support Vector Machines offer good methods in addition to existing homology searches for DNA repair protein identification and functional annotation. Most importantly, this study has uncovered relationships between the size of a genome and a genome's available repair repetoire, and offers a number of new predictions as well as a prediction service, both which reduce the search time and cost for novel repair genes and proteins.

## Background

The DNA in cells of living organisms continually suffers endogenous and exogenous damage. For example, cytosine will sometimes spontaneously change into uracil because of the loss of an amino group, and UV-A rays found in sunlight at the surface of the Earth cause DNA single-strand breaks, just two of the many documented sources of DNA damage [1,2]. Endogenous damage is more frequent and largely unavoidable [1]. In response to the many types of damage that DNA suffers, there are equally a myriad of methods to reverse the changes incurred. DNA repair is believed to exist in any organism with metabolic activity, and recent evidence suggests that even ancient bacteria from as many as tens of thousands of years ago was capable of DNA repair [3]. Of the many forms of DNA repair, nucleotide excision repair, or NER, is a critical repair system because of its ability to repair bulky lesions that consist of more than one nucleotide [1] and its complexity in utilizing at least 25 different polypeptides [4]. Another key mechanism is the mismatch repair system, which improves the error rate when copying DNA from one mistake per 10^7 ^nucleotides to one mistake per 10^9 ^nucleotides [5]. However, there are some subtopics of DNA repair, such as translesion DNA synthesis (TLS), which are still at a primitive level of understanding [2].

Much of the knowledge on DNA repair that has been accumulated is the result of biological experiments and clinical trials, and there exist only a few bioinformatics-based approaches to extract additional knowledge on DNA repair. One such approach is the Repair-FunMap, a functional database of proteins of the human DNA repair systems [6], which leverages a portion of its knowledge on the list created by Wood and his colleagues for annotation of human DNA repair genes [7]. The list created by Wood *et al*. provides accession numbers so that genes can be referenced electronically. Only recently have a few other repair-related analyses appeared utilizing bioinformatics, such as to identify sites of phosphorlyation [8] or study a particular gene [9].

The two aforementioned databases are limited to knowledge based on human repair pathways, and are largely the result of manual observation. However, with an increasing number of genomes being sequenced, manual observation for DNA repair patterns across all species is costly. For example, the Japanese medaka fish has 548 functionally "known" proteins but 24,113 proteins in its genome are still classified as "novel" in the March 2008 version of the ENSEMBL genome database [10], where novel in this context means that they cannot be mapped to any species-specific entry in several well known protein annotation databases. ENSEMBL's 2008 estimate of a combined 19,686 genes in the medaka genome, including genes with multiple protein transcripts, is in close agreement with other medaka documentation [11]. It would be useful to have a computational tool available to automatically identify potential DNA repair proteins in new genomes, to support or suggest further review of existing annotations, and to additionally characterize the function of new repair proteins. We have created a unique web-based tool to do such tasks (see Results:Web Service section for more).

Automated sequence analysis for determining the roles of proteins is not a new concept. There have been a variety of methods proposed, from simple to complex, for determining proteins of differing types. A simple method that uses only amino acid or dipeptide frequency was used to detect and classify histones [12]. Specific sequence patterns or profiles resulted in a subcellular localization predictor that outperformed a homology-based method [13]. Amino acid composition combined with periodicity is a technique competitive with other methods for predicting DNA- and RNA-binding proteins [14].

We hypothesize that using machine learning can yield predictions of unannotated proteins that are involved in DNA repair with high likelihood, and with improved reliability compared with existing and often-used homology searches. We further clarify that the objective of this paper is not to study any specific repair gene in a particular organism, but rather to establish that several general repair patterns exist in all organisms, to provide new computational tools for DNA repair research, to use those tools to identify more proteins involved in repair, and to convey the computational complexity of repair protein prediction analogous to its real world complexity.

We use a series of conceptually simple data transformation techniques incorporating combinations of primary sequence, predicted secondary structure, and homology search information to create feature vectors for input to a Support Vector Machine (SVM). We include homology information in our feature vectors since it is already established that homology search is useful for identification and classification of proteins (e.g. [13,15]). The transformation-based SVM experimental results are compared with independent BLAST [16] trials.

Using the Protein Data Bank [17] (PDB) and UniProt [18], two identification experiments are performed to distinguish between proteins that contain the Gene Ontology [19] (GO) categorizations "DNA Repair" and "nucleus" (see Datasets for details). Since using BLASTclust would introduce a bias, we use the program CD-HIT [20] for generating data subsets of increasing dissimilarity. Experiments are performed using originally obtained datasets as well as datasets clustered at 90% (non-redundancy) and 50% sequence similarity, the latter similarity threshold used due to software limitations. The area under the ROC curve (AUC) and the rate of true positives (TPR, or sensitivity) allowing no false positives, two statistics often used in data-based inference (c.f. [21,22]), as well as true positive rates allowing maximums of 1% and 5% false positives are used as the statistical metrics to gauge our experimental results. We choose 1% and 5% false positive rates because statistical tests typically use Type-I (false positive) error cutoff rates of 1% or 5% (c.f. [23]).

In the same fashion, we use the data transformations in DNA repair protein classification experiments. For these experiments, we use the 20 GO-based DNA repair pathway classifications and extract proteins from UniProt which have such classifications. Here, classification is the ability to distinguish between proteins belonging to a specific repair pathway and proteins not belonging to that repair pathway. Finally, based on the results of the repair protein identification experiments, we use both the disciminators resulting from machine learning and the generative model from homology to scan for the presence of DNA repair proteins in 31 vertebrate genomes available from ENSEMBL.

## Results

### Repair protein identification

**Figure 1:**
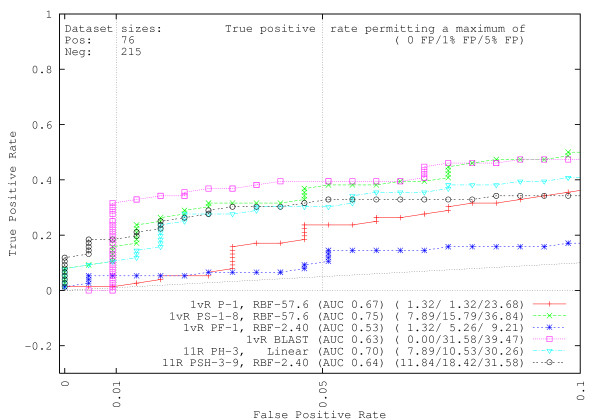
**GO-PDB identification experiments at 50% sequence similarity**. In each line at the bottom of the graph, the computational method (see Table 1 for abbreviations) used to identify DNA repair proteins is given. Methods other than BLAST used SVMs, and the value(s) of the primary sequence and (where applicable) secondary structure spectrum kernels, as well as experimentally optimal SVM parameters are listed after the transformation method (e.g., 11R PSH-3–8 means that method PSH used a primary sequence 3-spectrum kernel, a secondary structure 8-spectrum kernel, and homology with 11R cross-validation; see Methods for the 11R method). To the right of each method, AUC values and true positive rates when allowing maximums of no false positives (FPs), 1% FPs, and 5% FPs, respectively, are shown.

**Figure 2:**
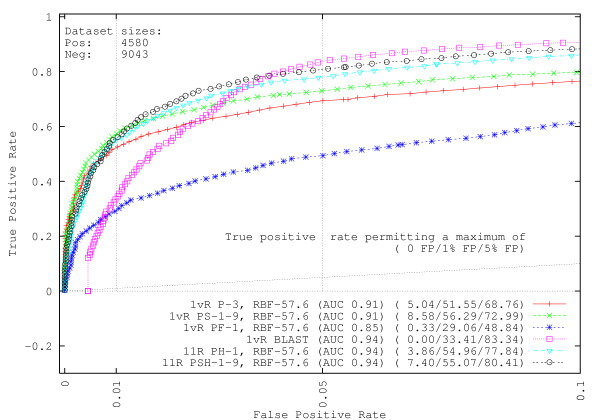
**GO-UniProt identification experiments at 50% sequence similarity**. Shorthand notation for computational methods is the same as in Figure 1. The SVM methods can find larger portions of the repair dataset while making few or no mistakes. Prediction performance is boosted through the use of secondary structure and homology.

We emphasize our prediction performance metrics by showing ROC curves in Figures 1 and 2 only up to a maximum of 10% Type-I error, adding faint vertical bars at the 1% and 5% Type-I error levels for easy visualization. The faint diagonals in the figures are the customary random guess performance lines. In the figures, each method name indicates the types of information used in the classifier, the training/testing dataset creation method, and, except for BLAST, spectrum kernel and SVM parameters used (see Methods). All methods are listed in Table 1 for quick reference.

**Table 1:** Data transformations

Method	Feature Vector Representation	Data Used
P	<*φ*_*s*_(*α*^*k*^, *Q*) >	Primary Sequence
PS	$<\phi_{s}(\alpha^{k_{1}},Q_{p}),\phi_{s}({\left\{C,E,H\right\}}^{k_{2}},Q_{s})>$	Primary Sequence and Secondary Structure
PF	$<\phi_{s}(\alpha^{k},Q),\phi_{f}^{C,E}(Q)>$	Primary Sequence and Frequency Priors
PH	<*φ*_*s*_(*α*^*k*^, *Q*), *φ*_*H *_(*Q*) >	Primary Sequence and Homology
PSH	$<\phi_{s}(\alpha^{k_{1}},Q_{p}),\phi_{s}({\left\{C,E,H\right\}}^{k_{2}},Q_{s}),\phi_{H}(Q)>$	Primary Structure, Secondary Structure, Homology
BLAST	BLAST	Homology

A summary of the six data processing methods used to identify and classify DNA repair proteins.

For experiments to identify repair proteins, Figure 1 and Table 2 respectively show the results of 5-fold cross-validation experiments using datasets extracted from PDB and clustered at 50% (Figure 1), 90% and 0% (unfiltered) similarity. Examining Figure 1, the results indicate that BLAST cannot detect DNA repair proteins without first making several mistakes, though the BLAST method exhibits a higher true positive rate at the 1% error rate for clustered data. Contrastingly, the SVM methods could all detect some portion of the DNA repair dataset without allowing false positives. Despite pre-experiment anticipation that inserting (amino acid frequency) prior knowledge into the feature vector would be successful (see Methods:Method PF for details), Method PF typically is the least successful. The addition of secondary structure (Method PS) considerably improves prediction results over using primary sequence information alone (Method P), as does the proposed feature vector transformation using primary sequence data and homology information (Method PH). Despite the reduction of training data by 60%, both of the one-versus-one-versus-rest methodologies described in the Feature Vector Methods section provide higher performance than the one-versus-rest primary spectrum transformation. In particular, the SVM combining primary structure, secondary structure, and homology information (Method PSH) provides the highest number of true positives when allowing no false positives to occur in clustered data. As can be seen in Table 2, when utilizing all of the original data in the PDB dataset, which would include multiple chains that may have identical or similar sequences, primary sequence data alone may be adequate to make an accurate DNA repair decision, as Method P can successfully detect 57% (318) of the DNA repair dataset proteins before making a false decision. Furthermore, allowing 1% Type-I error, the primary structure method can find 76% (425) of the DNA repair proteins. Generally speaking, we found that the SVM methods typically could complete detection of all proteins in the DNA repair protein dataset with a lower false positive rate than that of BLAST (graph and tables not extended to show this, though this is demonstrated by the AUC scores). Unfortunately, such false positive rates at 100% TPR using clustered data are unacceptable for any sort of inference application (more in Discussion section).

**Table 2:** Identification experiments using GO-PDB data

Seq. Similarity	Pos/Neg (% Pos)	Methodolgy	AUC	TPR-0	TPR-1%	TPR-5%
		P-1	0.67	1.32	1.32	23.68
		PS-1–8	0.75	7.89	15.79	36.84
50% (Figure 1)	76/215 (21%)	PF-1	0.53	1.32	5.26	9.21
		BLAST	0.63	0.00	31.58	39.47
		PH-3	0.70	7.89	10.53	30.26
		PSH-3–9	0.64	11.84	18.42	31.58

		P-3	0.68	12.28	14.04	30.70
		PS-1–9	0.77	5.26	7.89	41.23
90%	114/353 (24%)	PF-3	0.64	0.00	7.02	19.30
		BLAST	0.59	0.00	33.33	46.49
		PH-3	0.70	8.77	20.18	39.47
		PSH-3–8	0.74	15.79	19.30	36.84

		P-3	0.96	57.27	76.48	83.12
		PS-3–9	0.96	53.14	64.99	82.76
0%	557/1443 (28%)	PF-3	0.89	25.85	32.50	63.38
		BLAST	0.73	0.00	60.14	66.97
		PH-1	0.90	17.59	25.49	66.07
		PSH-3–8	0.91	39.50	55.30	71.10

The results (including Figure 1) of DNA repair protein identification experiments using proteins drawn from the Protein Data Bank containing the DNA repair Gene Ontology. "Pos/Neg" shows the respective numbers of repair proteins and nuclear non-repair proteins. Column meanings are similar to Figure 1. For each sequence similarity dataset, the top performing method for each metric is underlined (unless a tie exists in more than half of the classifiers).

It is important to establish whether the different methodologies have a statistically significant difference (the alternative hypothesis) in terms of their performance, because results may contain variance based on the selection of the test and training datasets [24]. Since we cannot assume that the AUC scores or other metrics used in this paper are normally distributed [25] (though this assumption is often allowed, e.g. [26]), we resort to the non-parametric Kruskal-Wallis test [27] to identify whether there is a statistically significant difference amongst the methods. As can be clearly understood from Table 2 and Additional files 1 and 2, we eliminate the results of Method PF when calculating the likelihood of classifier equality. For the unfiltered GO-PDB dataset, we used the 5 sets of AUC values from each fold of cross-validation and found a significant difference amongst the methods (P-value 0.0191). The result means it is statistically very unlikely that all classifiers used in this paper are equivalent on average, and therefore, we perform additional pairwise comparison of methodologies in Table 3. For pairwise comparison, we use both the parametric t-test which assumes data is normally distributed and the non-parametric Wilcoxon signed-rank test (c.f. [25,27]), and find that the conclusions are similar. Combining the information from Tables 2 and 3, we can say that there is a statisically significant improvement when using Methods P and PS to analyze GO-based DNA repair proteins in the unfiltered GO-PDB database. Results of Kruskal-Wallis tests for the remaining GO-PDB and GO-UniProt datasets are given in Additional file 3, including methodology pairwise comparisons.

**Table 3:** Pairwise comparison of classifiers.

Methodology	Test	P	PS	PH	PSH
PS	T	0.7153			
	W	0.8413			

PH	T	0.0844	0.1287		
	W	0.0952	0.1508		

PSH	T	0.1006	0.1705	0.7109	
	W	0.1508	0.2222	0.8413	

BLAST	T	**0.0094**	**0.0104**	0.2442	**0.0193**
	W	**0.0159**	**0.0159**	0.0952	0.0556

Using both the (parametric) t-test (T) and the (non-parametric) Wilcoxon Signed-Rank Test (W) on the original GO-PDB dataset, the probability that each pair of classifiers produces the same AUC score on average is listed. The results indicate that the various SVM formulations are not significantly different statistically, though there is a statistical contrast to BLAST (indicated in bold), and SVM formulations including BLAST result in lower probabilities of producing results identical to non-homology feature vector formulations. Bold values indicate the statistical difference is significant at the 0.05 confidence level and we can reject the null hypothesis that the classifiers are equivalent on average.

Figure 2 and Table 4 show similar experiments using data from the UniProt database. Experiments again show that the SVMs can detect some portion of the DNA repair dataset before making a false decision, in contrast to BLAST which unfortunately cannot detect any DNA repair proteins without first loosening its threshold and accepting some false positives. This is an unexpected result because as mentioned in the Datasets section, UniProt proteins are sometimes inferred from homology, and despite our filtering process to remove as many of these putative proteins as possible, we anticipated that some homologs would remain, and hence that BLAST would produce superior results. In terms of the AUC score, the SVM methods and BLAST produce similar results, though the SVM methods have higher rates of DNA repair protein identification when allowing no false positives or only 1% of false positives in the UniProt dataset. At the 5% Type-I error acceptance level, all of the methods except for the prior knowledge method (Method PF) produce approximately similar results. The additions of secondary structure information and homology again provide boosts in predictive power when data is clustered. The results shown in Table 4 for the identification experiments with the GO-UniProt data suggest that the optimal method is dependent on the amount of Type-I error tolerance and sequence similarity used. ROC curve figures for identification experiments performed at 90% and 0% similarity are provided in Additional file 1.

**Table 4:** Identification experiments using GO-UniProt data

Seq. Similarity	Pos/Neg (% Pos)	Methodolgy	AUC	TPR-0	TPR-1%	TPR-5%
		P-3	0.91	5.04	51.55	68.76
		PS-1–9	0.91	8.58	56.29	72.99
50% (Figure 2)	4580/9043 (34%)	PF-1	0.85	0.33	29.06	48.84
		BLAST	0.94	0.00	33.41	83.34
		PH-1	0.94	3.86	54.96	77.84
		PSH-1–9	0.94	7.40	55.07	80.41

		P-3	0.96	14.70	72.29	84.57
		PS-1–8	0.95	17.41	74.15	84.85
90%	11267/14257 (44%)	PF-3	0.86	3.83	37.13	50.30
		BLAST	0.97	0.00	47.56	92.36
		PH-3	0.97	20.13	67.81	84.49
		PSH-1–9	0.97	11.08	74.14	88.42

		P-3	0.98	23.40	82.09	91.12
		PS-3–9	0.97	24.23	81.38	89.63
0%	17828/19348 (48%)	PF-3	0.87	3.48	46.42	56.46
		BLAST	0.98	0.00	52.88	92.69
		PH-3	0.98	8.46	78.00	91.18
		PSH-1–9	0.98	4.31	80.45	92.06

The results (including Figure 2) of DNA repair protein identification experiments using proteins drawn from UniProt containing the DNA repair Gene Ontology.

### Repair protein classification

We performed classification experiments in the same manner as identification experiments by extracting proteins from the UniProt database which contain each of the Gene Ontology DNA repair subtypes (Kruskal-Wallis analysis is given in Additional file 3). Tables 5, 6, 7 show the results of classification experiments for identification of proteins involved in double strand break repair (GO ID:0006302), nucleotide excision repair (GO ID:0006289), and regulation of the DNA repair process (GO ID:0006282). Results of additional repair pathways are given in Additional file 2. Each table shows the results of the top performing feature vector methodology (regardless of detailed parameters) and BLAST, at the same sequence similarities as in identification experiments. The results further underscore the difficulty of the repair identification and classification problem, as no method is superior in all evaluation metrics.

**Table 5:** Classification: Double Strand Break Repair

Seq. Similarity	Pos/Neg (% Pos)	Method	AUC	TPR-0	TPR-1%	TPR-5%
		P	0.82	9.77	24.71	40.23
		PS	0.89	25.86	54.02	66.67
50%	174/1656 (10%)	PF	0.80	9.77	17.82	35.63
		BLAST	0.88	15.52	64.37	73.56
		PH	0.83	5.75	17.82	47.70
		PSH	0.89	1.72	25.29	64.94

		P	0.92	16.92	55.64	71.05
		PS	0.94	27.44	69.92	77.82
90%	266/4379 (6%)	PF	0.90	27.44	46.62	67.29
		BLAST	0.92	13.53	76.69	83.08
		PH	0.89	0.38	19.92	40.23
		PSH	0.93	7.14	34.59	67.67

		P	0.94	26.65	63.19	75.82
		PS	0.94	34.62	73.90	83.24
0%	364/6983 (5%)	PF	0.91	26.65	58.24	69.51
		BLAST	0.93	0.00	78.30	86.81
		PH	0.87	2.47	10.71	29.12
		PSH	0.93	2.75	29.12	60.71

Experiments using Double Strand Break repair (GO:0006302) as the type of protein to identify. "Pos/Neg" shows the number of DSB repair-related and non-related proteins for a particular sequence similarity.

**Table 6:** Classification: Nucleotide Excision Repair

Seq. Similarity	Pos/Neg (% Pos)	Method	AUC	TPR-0	TPR-1%	TPR-5%
		P	0.90	37.47	50.69	66.12
		PS	0.91	35.54	56.75	71.07
50%	363/1467 (20%)	PF	0.86	22.04	41.60	56.47
		BLAST	0.95	53.99	84.85	88.43
		PH	0.94	2.75	43.25	79.89
		PSH	0.95	4.68	56.75	87.88

		P	0.98	81.58	87.55	92.23
		PS	0.98	80.23	88.83	92.45
90%	1325/3320 (29%)	PF	0.88	25.89	39.77	63.77
		BLAST	0.98	0.00	94.19	97.13
		PH	0.98	4.15	75.02	95.25
		PSH	0.98	1.43	84.53	96.45

		P	0.98	37.65	89.27	94.16
		PS	0.98	65.19	90.41	93.16
0%	2106/5241 (29%)	PF	0.88	21.13	39.55	54.37
		BLAST	0.97	0.00	90.03	96.96
		PH	0.97	3.23	64.15	82.15
		PSH	0.98	11.49	79.49	91.36

Experiments using Nucleotide Excision Repair (GO:0006289) as the type of protein to identify. "Pos/Neg" shows the number of NER-related and non-related proteins for a particular sequence similarity.

**Table 7:** Classification: Regulation of DNA Repair

Seq. Similarity	Pos/Neg (% Pos)	Method	AUC	TPR-0	TPR-1%	TPR-5%
		P	0.97	57.02	92.98	94.74
		PS	0.98	87.72	93.86	95.61
50%	114/1716 (6%)	PF	0.96	51.75	92.11	93.86
		BLAST	0.96	85.96	94.74	94.74
		PH	0.99	50.88	93.86	97.37
		PSH	0.99	76.32	93.86	97.37

		P	0.98	62.64	94.83	97.13
		PS	0.99	90.80	95.40	97.70
90%	174/4471 (4%)	PF	0.97	52.30	93.68	94.83
		BLAST	0.99	82.76	97.13	97.13
		PH	0.99	62.07	97.13	98.28
		PSH	0.99	83.33	95.98	97.70

		P	0.99	69.70	97.35	98.48
		PS	0.99	88.26	96.97	99.24
0%	264/7083 (4%)	PF	0.99	36.74	95.83	97.35
		BLAST	0.99	21.97	98.48	98.48
		PH	0.99	36.74	98.86	99.24
		PSH	0.99	80.68	98.11	99.24

Experiments using "Regulation of DNA repair" (GO:0006282) as the type of protein to identify. "Pos/Neg" shows the number of regulation-related and non-related proteins for a particular sequence similarity.

For DSB repair, inclusion of secondary structure produces the best classifier when not accepting false positives, regardless of sequence similarity. The power of homology search is evident when we allow a few false positives to occur, as BLAST produces the best true positive rates at 1% or 5% Type-I error levels. Method PS also produces good true positive rates when allowing a small number errors to occur. For NER, the repair pathway discussed in the Introduction, BLAST is clearly the classification method of choice, producing top results in terms of all four metrics for most of the sequence similarity datasets we created. The only drawback to BLAST is that it suffers from a problem similar to the identification problem, where it often cannot detect NER proteins without first admitting a few false positives. SVM Methods P and PS fill that niche by detecting large portions of the NER dataset without making a wrong decision. For experiments classifying proteins related to regulation of the repair process, a combination of primary sequence, secondary structure, and homology produces the top AUC scores as well as almost 100% true positive rates at 5% Type-I error acceptance. Experiments using only primary and secondary structure produce top TPR rates when allowing no false positives.

The classification results show how each method has its strengths and weaknesses. Nucleotide Excision Repair, a critical repair system, is best detected through BLAST, whereas proteins related to regulation of the repair process are best found via SVMs including either secondary structure information, homology, or both. It is difficult to decide a superior technique for Double Strand Break repair, because the superior method depends on the metric used. For future large-scale applications to scan and classify genomes, an expert in each repair pathway would be able to suggest the best criteria for optimal prediction.

### Genome scanning

As the first of three main objectives, we investigated a relationship between the size of a genome as reported by ENSEMBL (version 48, December 2007), and the number of DNA repair proteins we detect in its resulting protein transcript. We chose the GO-based PDB repair and non-repair datasets to use as our training data, because PDB data has been experimentally observed, and because the highest percentage of repair protein identification allowing no false positives to occur was obtained using the PDB dataset.

**Figure 3:**
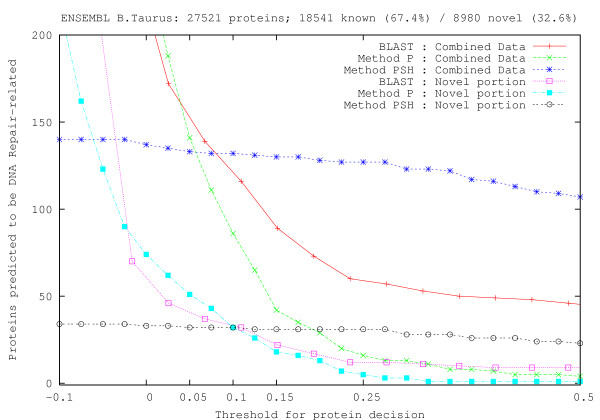
**Genome scan of the cattle genome Bos Taurus**. A plot of the number of repair proteins as a function of methodology threshold. Despite different technique formulations, both BLAST and SVM (Method P) show an exponential decrease in the number of proteins likely to be related to DNA repair as the decision threshold is tightened. The BLAST approach has had e-values adjusted to fit in the graph. At a Method P threshold of 0.151 which resulted in no false positives and 57% true positives in GO-PDB identification experiments, several proteins of unknown function are predicted to be DNA repair related.

First, we scan a single genome divided into its known and novel protein transcripts using Method P, Method PSH, and BLAST, and evaluate the number of potential repair proteins as a function of detection threshold (SVM: score; BLAST: e-value). Thresholds for analysis of results are chosen based on the GO-PDB identification experiments: for Method P, we select the thresholds 0.151 and 0.001, respectively corresponding to 0 FP/57.2% TP and 0.1% FP/64.6% TP; for BLAST we use the threshold 10^-3 ^which resulted in 1.7% FP/63.3% TP in identification experiments, and is often used as a threshold in other homology-based research (e.g. [12,15]). Figure 3 shows a graphical result from scanning the cattle genome Bos Taurus, where only the novel and combined protein dataset scan results are shown. To overlay with SVM results, e-value thresholds used with BLAST are adjusted to fit in the graph using the conversion

$$f(\theta_{B})=L+\log(\theta_{B})*\frac{M_{svm}-m_{svm}}{\log(M_{blast})-\log(m_{blast})},$$

where *θ*_*B *_is the BLAST threshold used for evaluation, *L *is the coordinate of the left end of the graph, *M*_*svm *_is the maximum SVM threshold used, *m*_*svm *_is the minimum SVM threshold used, *M*_*blast *_is the maximum BLAST threshold used (small e-value), and *m*_*blast *_is the minimum BLAST threshold used (large e-value). As can be seen from the graph, the number of predicted repair proteins decreases exponentially as a function of the threshold, for both BLAST and Method P. The inclusion of secondary structure information appears to induce a number of false positives, which is an unexpected result given the merits of secondary structure observed in identification and classification experiments. The result of the scan suggests that there are still a number of repair proteins to be experimentally observed and annotated into databases, because a number of proteins are detected in novel portions of the cattle genome at thresholds above (for BLAST, below) the thresholds stated at the beginning of this section which produce, if any, minimal false positives.

**Figure 4:**
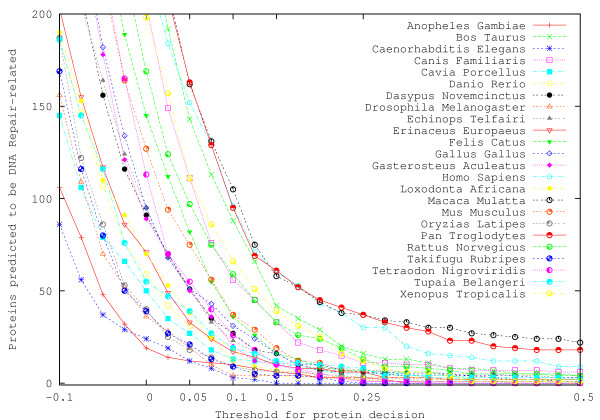
**Genome scans of 31 organisms**. A plot of the number of repair proteins detected in 31 vertebrate genomes as a function of Method P threshold. In all organisms, the predicted number of repair proteins decays exponentially with increasing detection threshold. Several genomes have been removed from the figure to prevent genome names from overlapping the results. At thresholds greater than 0, all organisms are predicted to have proteins related to DNA repair, consistent with the findings in [3].

We apply the same process to the scanning of an additional 30 genomes in ENSEMBL, and find that there is a similar pattern overall in most genomes, as seen in Figure 4. In the case of known human repair proteins, the Method P threshold of 0.151 corresponds to detection of 49 DNA repair proteins, while the threshold of 0.001 produces 184 detections. A BLAST threshold of 10^-3 ^results in 114 detections. The result is consistent with identification experiments where the higher SVM threshold produced a lower number of true positives with no false positives, and the minimally positive threshold produced relatively few false positives but with more true positives (see "Finding new candidates" section for examples of true positives). More importantly, the BLAST and averaged SVM results concur with experimental observation in which at least 138 DNA repair proteins are known to exist in humans [1,7]. Additional scanning of the novel human dataset yielded 11 potential proteins via SVM and 16 potential proteins via BLAST (threshold 10^-2^), which is in agreement with the unknown status of existence in several human repair pathways [1]. This experiment interestingly resulted in clustering of humans, chimpanzees, and monkeys. At the threshold of 0.151, the results also cluster together the four-legged species cattle, dog, frog, and rat.

**Figure 5:**
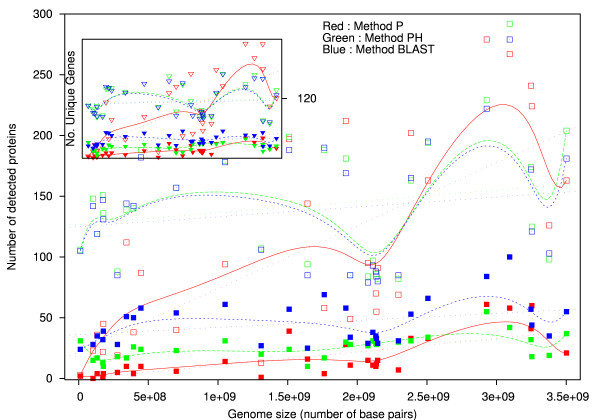
**Correlation of genome size to number of repair proteins and repair genes**. The outer plot shows the relation between the size of a genome (horizontal axis) and the number of predicted repair-related protein transcripts in that genome (square points). Dual thresholds are used for Methods P (red), PH (green), and BLAST (blue), where one threshold produced no false positives in identification experiments (lower three curves), and another threshold produced a small percentage of false positives while admitting more true positives (upper curves). In the small inner plot, we reduce the number of detected proteins to the number of unique genes (vertical axis), and again plot versus the genome size (triangular points). The following thresholds are used for each detection technique: Method P: 0.001/0.151; Method PH: 0.001/1.015; BLAST:10^-3^/10^-9^. Data is smoothened by using bezier curves. Correlations between the size of a genome and the number of repair transcripts are listed in Table 8 for both of the Method P thresholds used. Though the tighter threshold dataset of the inner plot visually appears to be approximated by a constant for methods PH and BLAST, the slope of the approximation is positive.

To answer the question as to whether or not there exists a correlation between the size of a genome and the (predicted) number of repair proteins, in Figure 5, we plot the size of each of the 31 genomes against the resulting number of repair proteins, using Methods P, PH, and BLAST. From our predictor, we additionally extracted the number of unique genes (sub-plot), to analyze the results in terms of both protein transcripts and originating genes. Data is smoothened using Bezier curves to convey general trends about the data. In Figure 5 and its sub-plot, we tested thresholds corresponding to the basic decision nature of the technique (10^-3 ^for BLAST and 0.001 for SVM methods), as well as thresholds (10^-9^; 0.151) which produced few (BLAST) or no (SVM) false positives in identification experiments.

From the figure, we can see a very small increase in the number of protein transcripts as the length of the genome grows, indicated by additional dotted linear approximations to the data. This trend is consistent through all three methods, though Method P produces a result with a larger slope as a result of its inability to find proteins in several smaller genomes such as *S. Cerevisiae *and *Drosophila Melanogaster*. The smaller figure located within Figure 5 shows a plot of the genome size versus the number of unique genes, and results here suggest that the number of fundamental genes involved in DNA repair is fairly consistent through many species, with a very minimal positive slope and some small degree of fluctuation among species of similar genome size. Though we are not directly implying a linear relationship between the size of a genome and its number of repair genes/transcripts, the linear approximations all being positively sloped on the order of 10^-9 ^suggest a non-negative and non-constant relationship, along with non-zero intercept values suggesting that there are some basic repair genes present in all organisms, a suggestion that is consistent with [3]. Counting the known and unknown status of over 150 repair genes listed in [1] which includes humans and four other species, and assuming that some portion yet not all unconfirmed gene statuses in [1] will turn out to have repair genes, then these findings of a slow increase in repair genes/transcripts in relation to a genome size are consistent. For repair protein transcripts, we can think of our two thresholds as upper bounds (a few false positives) and lower bounds (no false positives), and we estimate that the true nature of the number of protein transcripts is a slowly-increasing relationship lying somewhere in between the two bounds. Datapoints in the outer plot of Figure 5 again confirm the consistency of the result with evolution, as humans, chimpanzees, and monkeys result in points on the right side of the graph in the cluster above the curves, and animals such as hedgehogs, shrews, and armadillos are clustered together in the middle slightly below the curves.

We scanned previous versions of ENSEMBL (versions 43 and 46) to validate the consistency of our result, and found relatively little change in the number of predicted repair proteins. Table 8 shows the correlation between the size of a genome and number of repair protein transcripts we predict it to contain. As shown in the table, the correlation is fairly strong, especially in the latest revision of the ENSEMBL database used.

**Table 8:** Correlation between size of a genome and number of DNA repair proteins

ENSEMBL version	SVM threshold	Pearson correlation	p-value	Spearman correlation	p-value
v43	10^-6^	0.7165	5.8e-06	0.7269	3.6e-06
v46	10^-6^	0.6876	1.9e-05	0.7252	3.9e-06
v48	10^-6^	0.7206	4.8e-06	0.748	1.3e-06

v43	0.151	0.6776	2.8e-05	0.7660	5.1e-07
v46	0.151	0.6730	3.3e-05	0.7661	5.0e-07
v48	0.151	0.7000	1.1e-05	0.7974	7.9e-08

The correlation is based on scanning of 31 vetebrate genomes in ENSEMBL using Method P. Scan results for a minimally positive threshold reflect the basic decision nature of the SVM algorithm. A threshold of 0.151 reflects the threshold needed to obtain a maximum true positive rate while allowing no false positives, and hence serves as a lower bound and reliable indicator of the positive correlation between genome size and number of repair proteins. ENSEMBL versions 43, 46, and 48 were released in February, April, and December 2007, respectively.

After combining predicted novel and known proteins in each genome, we investigated the extent to which multiple methods overlapped in their detections. Using data from Homo Sapiens, Mus Musculus, Bos Taurus, Gallus Gallus, and Drosophila Melanogaster, sizes of overlapping protein identifier sets are given in Table 9. It is clear from the results that the inclusion of homology influences the Method PH predictor, as the numbers of detected proteins are similar to BLAST, and overlaps are large. These results also indicate that despite the different approaches of Method P and BLAST, several proteins exhibit multiple features that clearly distinguish them as DNA repair-related. For example, the transcripts ENSBTAT00000000276 and ENSBTAT00000003559 in Bos Taurus, corresponding to DNA polymerase beta and a DNA lyase (c.f., [1]), produce high scores in both methods.

**Table 9:** Overlap in detection datasets.

Species	Prot-P	Prot-PH	Prot-BLAST	P-PH	P-BLAST	PH-BLAST
Bos Taurus	241	174	172	12	13	162
Drosophila Melanogaster	36	119	119	2	2	112
Gallus Gallus	94	179	178	10	11	165
Homo Sapiens	224	125	121	17	17	114
Mus Musculus	126	98	103	5	7	90

The table shows the overlap amongst the sets of protein transcript IDs for proteins predicted to be involved in DNA repair for five species. In the second through fourth columns, Prot-X is the number of detected proteins per method X, and the fifth through seventh columns show the overlap between each pair of methods.

Another interesting trend we found is shown in Table 10. The table shows the scanning methodology, scan threshold, total number of detected repair protein transcripts in both novel and known portions of all genomes, total number of unique genes, and the ratio of unique genes to transcripts. As mentioned in the Methods section, it is known that a small number of genes are involved in multiple repair activities. The list created by Wood *et al*. [7] lists 86% of the genes to have a single repair function. Our results, averaged over all 31 species and including Method PS, estimate that 83% (std. dev. 2.9%) of genes have a single repair function, which is in close agreement with Wood *et al*.'s human documentation. The values of 86% and 83 ± 3% are also close to the ratio in the medaka fish mentioned in the introduction, as the medaka ratio of genes to transcripts is 80%.

**Table 10:** Relationship amongst detected transcripts and unique genes

Methodology	Threshold	No. Transcripts	No. Unique Genes	% Unique Genes
P	0.001	3330	2851	85.6%
	0.151	555	461	83.1%

PS	0.001	67393	56145	83.3%
	0.670	4144	3603	86.9%

PH	0.001	4403	3558	80.8%
	1.015	796	689	86.6%

BLAST	10^-3^	4302	3492	81.1%
	10^-9^	1419	1123	79.1%
Average Percentage of Unique Genes				83.3%
Standard Deviation				2.9%

The total number of detected protein transcripts and total number of resulting unique genes is given for each method using two thresholds derived from GO-PDB identification experiments. The average of 83.3% is in close agreement with findings in [7].

We additionally analyzed the collection of datasets produced by Method P to confirm to what extent they are similar in terms of their sequences. From the 3330 transcripts produced (Table 10), clustering down to 50% similarity using CD-HIT resulted in 1774 proteins. This diversity again confirms the challenge in building a single superior repair protein predictor. We then further took the (human/chimpanzee/monkey), (cow/dog/frog/rat), and (hedgehog/armadillo/opossum/shrew) clusters that we discussed above, and searched for the presence of identical proteins, where identical was thresholded as each of 100%, 99%, or 98% sequence similarity. The results in Table 11 show that a number of repair proteins in the primate cluster and four-legged species cluster are completely identical, and that more than 10 repair proteins per cluster are over 98% similar. For the third cluster which includes a number of animals that live in desert-like environments, there are no overlaps in the known portions of detected repair proteins, though the total number of detected known proteins in this cluster is less than 5 for each of Methods P, PH, and BLAST. In the case of novel proteins, a few overlaps are found for nearly-identical repair protein sequences. Detailed overlap results for each of the species in the three clusters can be observed in Additional file 4.

**Table 11:** Multi-genome overlaps

		Sequence similarity					
		100%		99%		98%	
Methodology		Known	Novel	Known	Novel	Known	Novel
P	Cluster 1	1	4	3	25	5	47
	Cluster 2	1	0	6	0	10	1
	Cluster 3	0	0	0	0	0	1

PH	Cluster 1	6	7	10	36	14	56
	Cluster 2	6	0	19	2	25	3
	Cluster 3	0	0	0	5	0	6

BLAST	Cluster 1	6	7	9	35	13	54
	Cluster 2	7	0	20	2	26	3
	Cluster 3	0	0	0	5	0	6

Using the clustering software CD-HIT, the number of identical proteins (sequences) found in novel and known portions of multiple genomes is listed when thresholding "identical" as each of 100%, 99%, or 98% sequence similarity. The clusters are as follows:

Cluster 1: Human, Chimpanzee, Monkey

Cluster 2: Cattle, Dog, Frog, Rat

Cluster 3: Hedgehog, Armadillo, Opossum, Shrew

### Finding new candidates

We examined the outputs of our genome scans to identify what types of proteins were being detected, and as our second main objective, to use this information to ascertain how well they can identify novel repair candidates. A number of high scoring proteins from well-studied genomes are listed in Table 12 and elaborated upon here. DNA glycosylases, which are important in the BER pathway [1,28], produced high scores and were found in multiple genome lists. The predictors also identified a number of DNA polymerases [1], as well as Rad51 proteins which are critical to the homologous recombination pathway [1,7,29,30]. Tdp1, whose failure has been linked to neurodegenerative disease [31], is a critical participant in repairing DNA damage [32] and is being investigated for its anticancer activity [33].

**Table 12:** Examples of Repair Detections

Genome	Identifier	Methodology	Pred. Score	Description
Homo Sapiens	ENST00000339511	P	0.950	N-glycosylase
	ENST00000339310	P	0.809	DNA Polymerase kappa
	ENST00000382643	P	0.469	Rad51 Homolog
	ENST00000357382	BLAST	1*e*^-167^	Tdp1
	ENST00000354383	BLAST	6*e*^-51^	DNA glycosylase

Mus Musculus	ENSMUST00000112275	P	0.662	Uracil DNA-glycosylase
	ENSMUST00000112723	P	0.143	Rad52 Homolog
	ENSMUST00000021594	BLAST	1*e*^-167^	Tdp1

Examples of detections in Homo Sapiens and Mus Musculus for proteins known to be involved in DNA repair and that are predicted via Method P or BLAST. Protein transcript identifiers are given in ENSEMBL format.

These types of proteins all have high scores above the thresholds that produced no false positives in identification experiments, and next we focus on the high-scoring proteins existing in novel datasets. Table 13 lists a handful of proteins that our predictor strongly suggests to be related to DNA repair. We are hopeful that these results can be either confirmed or disconfirmed in future laboratory experiments. In Additional file 5, we include high-scoring candidates for all 31 genomes used in this report.

**Table 13:** Novel proteins predicted to be repair-related

Genome	Protein ID	Chromosome	Base Pair Locations
Homo Sapiens	ENST00000383825	3	9767733–9783421
Rattus Norvecigus	ENSRNOT00000000872	12	43520629–43529526
Gallus Gallus	ENSGALT00000005973	22	2794396–2802220
Pan Troglodytes	ENSPTRT00000037442	8	39101917–39137673
Oryzias Latipes	ENSORLT00000022300	24	20404058–20406887

Examples of Method P high-scoring repair candidate proteins for several species are given. We list the organism, ENSEMBL protein identifier, and chromosome location. A full list of candidates from all 31 genomes used in this report is available as Additional file 5.

### Web Service

Based on the high performance obtained using original datasets of known repair proteins, as our third and final objective, we have implemented a web service which uses the processing techniques in this report to predict whether or not a protein is DNA repair related. The INTeractive dna REPair prEDiction server, or INTREPED, is a free service for research use, and we hope that it can assist researchers around the world working on genomes which are either unannotated, newly sequenced, or under revision. Though the human genome has largely been mapped out and those genes involved in DNA repair in humans are mostly known [1,6,7], there still exist many more genomes to be annotated, and this is where we believe INTREPED can be a valuable research tool.

**Figure 6:**
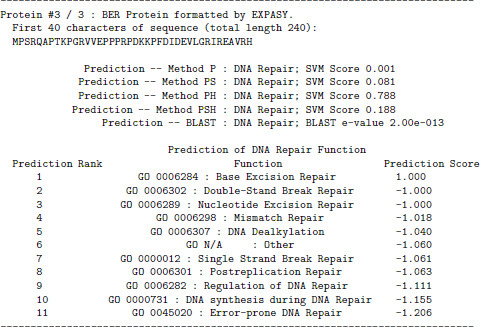
**INTREPED web server output**. An example of the email response produced by the INTREPED web server. Email responses include secondary structure analysis.

The INTREPED web server is accessible from the "Prediction Servers" page at . The user simply inputs a series of FASTA-format sequences. Users have two options for processing: they can immediately obtain a result in their web browser using Methods P, PH, and BLAST, or they can submit a request to a queue along with an e-mail address to return results, and analysis will additionally include use of secondary structure (Methods PS and PSH) which was shown to be effective in identification experiments. In Table 14 we list the expected prediction accuracy of each methodology and how to intepret the results. An example of an e-mail response from INTREPED is shown in Figure 6.

**Table 14:** Web Server Accuracy

Methodology	Threshold	True Positive Rate	False Positive Rate
P-3	0.001	64.6%	0.1%
	0.151	57.3%	0

PS-3–9	0.001	72.3%	2.4%
	0.670	53.1%	0

PH-3	0.001	65.0%	1.7%
	1.1015	7.9%	0

PSH-3–8	0.001	61.6%	1.7%
	0.727	39.5%	0

BLAST	10^-3^	63.4%	1.7%
	10^-9^	60.1%	0.7%

Metrics for interpreting the results produced by the INTREPED web server. For each of the five methods INTREPED employs, thresholds necessary for basic decision making and maximum TPR-0 rates are given. Each method and threshold pair provides the true positive and false positive rate based on identification experiments.

If the protein is predicted to be DNA repair related, an additional prediction using specialized per-pathway models occurs for classification, and resulting scores rank the repair protein's most likely functions.

INTREPED uses a rapid spectrum kernel implementation (see Additional file 6) to ensure quick prediction response and support large queries. The binary frequency kernel (Method PF) is not used in INTREPED, as its performance in its current design is not warranted.

## Discussion and Conclusion

When scanning genomes, the number of proteins predicted to be DNA repair-related as a function of the prediction threshold follows an exponential distribution (Figures 3 and 4). Despite the lower performance of Method PF, its intuition is validated by this result. If the amino acid distribution of DNA repair proteins was random, the resulting feature space would be uniformly populated, and we would be able to see a linear gradation in the number of predicted proteins as a function of prediction threshold. Since this is not the case, we are led to believe that DNA repair proteins are richer in some amino acids and tripeptides, and that we can rapidly segregate those proteins that do not have a similar distribution of tripeptides. For example, in the GO-based PDB dataset that we created (557 proteins), the tripeptides LEK, GAE, and ARK appear 314, 209, and 195 times, respectively. In the GO-UniProt dataset (17,828 proteins), the tripeptides ALA, EAL, and LLE occur respectively 9501, 8379, and 7511 times. Both datasets indicate that alanine, leucine, and glutamic acid are abundant in repair proteins. However, subsequences of length 4 or greater occurred with low frequency relative to the sizes of the datasets (see below for additional comments).

The size of a genome has been shown to have a high correlation with the number of repair protein transcripts. High Pearson correlations (Table 8) suggest that this approximation is reasonably accurate, and high Spearman coefficients suggest that even if the true nature of the number of repair proteins as a function of genome size is not linear, it is well correlated to a non-decreasing function of genome size. Since it is known that growth temperature affects metabolic network structure [34], and DNA repair is thought to exist in any organism with metabolic activity [3], comparison of this study to a similar work using archea or bacteria would further clarify the relationship between evolution, environment, genome size, and repair ability.

It remains an open question whether or not there is one ideal prediction method that can adapt to all forms of repair. To this end, we have shown statistically that not all methods are equivalent, which suggests that it may be possible to build a better sequence-based predictor specifically for DNA repair protein detection and recognition. We have not discussed the possibility that our source datasets used in classifier comparison and genome scanning may contain improper Gene Ontology labelings. As Dietterich has pointed out, if some small percentage *η *of our source data is in fact mislabeled, then our results herein can be have no less than an error rate of *η *[24], and the ideal classifier will remain elusive. Therefore, though our datasets have been selected reasonably, it also remains an important goal in research to establish a "golden standard" dataset for DNA repair protein prediction.

The methods presented herein are successful at recognizing and classifying DNA repair proteins, but many extensions and biologically significant improvements are still possible. First, when the number of sequences available is small as in the case of the PDB identification dataset filtered to 50% similarity, higher spectrum kernels may not be as successful because of a lack of data combined with RBF kernels. This is evident by the fact that we performed an experiment using unfiltered PDB data and Method P set to 4-spectrum, and found no improvement in results (data not shown). Hence filtered data would have an even more sparse representation that is less efficient, and therefore, it is worth pursuing a mathematical formulation for the optimal values of the primary and secondary spectrum kernels, as well as the RBF kernel parameter *γ*, since these values work as an interconnected system affecting prediction performance. When the feature space is sparse, the RBF kernel may indeed be less effective than small degree polynomial kernels (e.g., linear kernels sometimes providing better performance than RBF kernels as shown in Figure 1). Considering these factors, development of a customized kernel method for further improved identification and classification of repair proteins is a topic that will be pursued.

Second, guided by the intuition of Method PF, Principal Component Analysis as a method for extracting the most significant polypeptides is likely to improve results. Similar to the preceeding discussion, when the feature space is sparse because of a lack of data or a lack of frequently occurring features, a reduction in dimensionality would project the sparse space into a more clustered space, which could then be used by the often-used RBF kernel more effectively.

Third, the use of sequence motifs in feature vectors would utilize expert knowledge and create better classifiers (c.f., [13]). In preliminary work, we performed multiple sequence alignments of DNA polymerases, but were unable to find applicable results. Another previous multiple alignment for analysis in DNA repair has been shown to produce little overlap [1]. As a result, sequence motif classifiers for specific repair pathways are likely to be more successful when based on protein domains rather than on global sequence alignments. Yet, creation of a bit string vector representing the presence or non-existence of specific domains is likely to be difficult to apply to detection, again because of sparse representation. It was shown in the Results section that no particular method was superior for all classification results. Hence, to build improved applications for repair protein annotation, it may be useful to build per-pathway servers that include per-pathway domains, and then create a general integrated meta-server which can incorporate many of the more specific but more accurate pathway servers. This "subserver-omniserver" approach is in use for subcellular localization prediction [35-39], and may be useful in future analyses of repair research that are still not understood, such as proteins related to transmission of damage detection signals or DNA infidelity tolerance (TLS) [2].

Finally, incorporating the proposed relationship between genome size and repair ability in such a way that it can improve the accuracy of the INTREPED web server is a future work.

## Methods

### Problem Definitions

We first consider the DNA repair protein identification problem: given the amino acid sequence of a protein, can we determine whether or not the protein is (directly or indirectly) involved in DNA repair? Formally, each query protein *Q *consists of a sequence *s*_1_*s*_2 _... *s*_*n*_, where *s*_*i *_∈ *α *for 1 ≤ *i *≤ *n*. *Q *is thus of length *n*, and *α *is our denotation for the standard 26-character alphabet excluding the symbols B, J, O, U, X, and Z.

Proteins using SVM frameworks undergo transformations as described below, whereas proteins tested with BLAST require no transformation. In both approaches a value (SVM score or BLAST e-value) is output. Comparing that output value to a threshold, we determine whether or not the technique calculated the protein to be involved in DNA repair. Since an e-value and a SVM score have different interpretations, we use a continuum of thresholds in order to obtain ROC curves and compare different techniques.

The classification problem is the following: given that a protein is known to be involved in DNA repair, identify *the *repair class (pathway) or type that it belongs to. This means that our consideration is for a single repair class, and though some proteins are known to be involved in multiple repair pathways, the majority of repair proteins are involved in a single repair pathway [1,7] or serve a single structural role in DNA repair, which justifies our approach. The classification problem is hence recast as an identification problem for one repair protein class versus all remaining protein classes, and can follow the same logic above.

### Datasets

To perform two identification experiments and a classification experiment, we built five protein databases. In order to ensure the reliability of our data sets, we first require that proteins from databases be catalogued by using the Gene Ontology annotation system. We also remove all proteins which contain the following keywords in their descriptions: putative, similar, possible/possibly, probable/probably, theoretical, and hypothetical.

For our identification experiments, we use two data sources: PDB and UniProt. The advantage of using PDB data is that the proteins have been experimentally observed and confirmed, and we can impose the constraint that X-ray crystallography data exists. Despite the possiblity of homologs, the advantage of using UniProt is its vast sequence repository, offering considerably more sequences for analysis, including research literature documentation for many proteins. Though Wood *et al*. has assembled an invaluable list of repair genes which can be referenced [7], these are restricted to humans, whereas our assembled databases do not include a restriction on the species.

Amongst the approximate 49,000 protein structures in the February 2008 version of the PDB, 245 of them matched the "DNA repair" GO label (GO ID:0006281), resulting in 557 sequences after extracting multiple chains. We also extracted 447 protein structures which have the "nucleus" GO label (GO ID:0005634), and remove those structures which already match the repair GO label, resulting in 1443 sequences. From the UniProt KB database, 17,828 DNA repair and 19,348 nuclear non-repair GO-based sequences were retrieved. The PDB sequences we use are comparitively shorter in length since they are divided into separate sequences for each chain, and some PDB structures contain multiple chains. The datasets (protein identifiers) used in this work are available as Additional file 7.

For our classification experiment, we used the 20 DNA repair pathway categories listed in Gene Ontology, and extracted proteins from UniProt with the matching ontology IDs. We chose the UniProt database over the PDB database because the PDB database currently contains data for only four major repair pathways: base-excision repair (GO ID:0006284), mismatch repair (GO ID:0006298), single strand break repair (GO ID:0000012), and double strand break repair (GO ID:0006302). For classification experiments, we require a minimum of 25 proteins in the pathway dataset, and as a result of insufficient data, some pathways are not used. Table 15 lists each of the categories used for classification experiments.

**Table 15:** Classification datasets

	Original (unfiltered) data				
			Statistical Properties		
Repair pathway	GO ID	No.Sequences	Ave. Length	Median Length	Std. Dev
Base excision repair	0006284	2624	276	251	134.7
DNA dealkylation	0006307	25	203	174	106.8
DNA synthesis during DNA repair	0000731	28	996	1103	581.5
Double-strand break repair	0006302	364	616	609	306.4
Error-prone DNA repair	0045020	46	1075	1077	46.8
Mismatch repair	0006298	1777	617	653	317.3
Nucleotide-excision repair	0006289	2106	732	685	261.6
Postreplication repair	0006301	28	449	350	350.7
Regulation of DNA repair	0006282	264	211	172	161.6
Single strand break repair	0000012	40	476	614	297.8
Other pathways	N/A	45	592	373	486.9

Total		7347	515	415	321.2

					
	Maximum 90% similarity				
			Statistical Properties		
Repair pathway	No.Sequences		Ave. Length	Median Length	Std. Dev

Base excision repair		1721	284	260	135.9
Double-strand break repair		266	603	611	309.8
Error-prone DNA repair		36	1077	1082	51.4
Mismatch repair		1020	710	768	274.9
Nucleotide-excision repair		1325	737	689	268.9
Regulation of DNA repair		174	205	168	153.4
Single strand break repair		25	490	403	316.9
Other pathways		78	579.8	373	539.1

Total		4645	534	516	322.7

					
	Maximum 50% similarity				
			Statistical Properties		
Repair pathway		No.Sequences	Ave. Length	Median Length	Std. Dev

Base excision repair		630	321	278	185.7
Double-strand break repair		174	656	655	343.6
Mismatch repair		468	718	710	302.6
Nucleotide-excision repair		363	684	633	360.6
Regulation of DNA repair		114	213	168	185.5
Other pathways		81	659	582	496.2

Total		1830	535	434	349.8

The number of proteins extracted from the UniProt database for each of the DNA repair pathways used in classification experiments is shown along with statistical properties regarding seqeuence lengths. Only the types of repair pathways which had sufficient data (minimum 25 proteins) for experiments are shown, along with a combined dataset of the types which did not have enough data.

For each protein in each dataset we used SSPro [40] to obtain a secondary structure role for each amino acid in the protein in question. The workstation version of SSPro is a three-class predictor, assigning either alpha helix, beta sheet, or "other structural function" for each amino acid in a query. As an example, if we create a short protein with primary sequence RSYMMLDKF, SSPro will return the secondary structure CCCEEHCCC.

### Data Division Technique

In data-based inference experiments, performance is often measured by using cross-validation, and in particular, one-versus-rest (1vR) cross-validation. In such a data division scheme (let us assume the division is *f*-fold), one portion (size $\frac{1}{f}$) of the data is set aside for performance evaluation (test data) while the remaining *f *- 1 portions are used for training data, and the process is repeated *f *times using different test and training data each time. Here, we propose one-versus-one-versus-rest (11R) cross-validation. The reason for this methodology is homology and will be explained in further detail in the Feature Vector Methods section below. 11R differs from the 1vR technique in that for *f*-fold validation, one portion is still set aside for evaluation, but instead of *f *- 1 portions of data for training, only a single portion is used for training, and the *f *- 2 remaining portions are used as a reference homology database for querying training and test data. The amount of training data is reduced by $\frac{f-2}{f-1}$. The end goal of the 11R technique is to combine homology and sequence data in an unbiased way and obtain a realistic estimate of method performance.

### Support Vector Machines and Feature Vector Methods

#### Support Vector Machines

We consider six methods for processing proteins in question. The methods are summarized in Table 1 for quick reference, and are explained in detail here. The first five of the methods feed transformed feature vector data into a SVM to arrive at a decision; the sixth requires no transformation. In short, SVMs derive decision or regression functions by solving a quadratic programming problem. For decision problems, the resulting decision function is used to test whether a new piece of data belongs to a particular class or not. They have been used in many decision problems [13,14], are well documented [41,42], and have been shown to outperform other deterministic techniques such as artificial neural networks or standalone principal component analysis [43,44].

The SVM implementation we use is SVM^*light *^[45], and the only parameter of the software that we manually set is the gamma value (*γ*) of the radial basis function (RBF) similarity metric. The RBF kernel measures the similarity of two feature vectors **x **and **z **using the function $K(x,z)=exp(-\frac{||x-z|{|}^{2}}{2\gamma^{2}})$, and the *γ *parameter controls the learning balance between possibly over-fitting (low *γ*) and over-generalizing (high *γ*). The effect of this parameter on prediction performance is shown in the Results and elaborated upon in the Discussion section.

#### Feature Vector Methods

The first method for creating a feature vector from a protein sequence is to simply count the number of occurrences of each amino acid in the protein and divide the counts by the length of the protein. Simple amino acid frequency was successful in identifying and classifying histones [12]. This counting scheme can be generalized to include polypeptides, and is called the *k*-mer string kernel or *k*-spectrum kernel [42,46], which we denote by *φ*_*s*_(). Normalization in the polypeptide case is the result of dividing the number of occurrences of each *k*-mer by the total number of possible *k*-mers (= *n *- *k *+ 1, where *n *is as defined in the Problem Definition section). Resulting vectors are of size 20^*k*^. Considering all possible subsequences of length *k *in the query sequence *Q *using symbols in *α*, we write feature vectors using this transformation (referred to as Method P) as

**v **= <*φ*_*s*_(*α*^*k*^, *Q*) >.

We use tripeptides (*k *= 3) in addition to amino acid frequencies in this paper. Dipeptides were considered in an unpublished preliminary work [47]. We can extend on Method P by including secondary structure data as well. As explained in the Datasets section, we use the software tool SSPro to predict the secondary structure role of each amino acid in the query protein. We then consider large length (*k *= 8 or *k *= 9) spectrum kernels on the secondary structure information. The primary sequence and secondary structure spectrum kernels are joined, producing feature vectors of the form

$$v=<\phi_{s}(\alpha^{k_{1}},Q_{p}),\phi_{s}({\left\{C,E,H\right\}}^{k_{2}},Q_{s})>,$$

where *k*_1 _and *k*_2 _are the spectrum kernel lengths for the respective primary and secondary sequences *Q*_*p *_and *Q*_*s*_, and C, E, and H represent the SSPro output indicating alpha helix, beta sheet, or "other structural function". This transformation is referred to as Method PS.

Notwithstanding evolution, the role of DNA repair is to preserve the information coded in a genome, and it therefore seems reasonable that DNA repair proteins are richer in some amino acids that account for their stable function than in other types of proteins. As a result, if we have a database of known DNA repair proteins, we can scan it for the frequencies of each type of amino acid, and use this information to determine if a query protein not belonging to the known database has a similar percentage of each type of amino acid. We need not consider all amino acids, especially those which are infrequent in the reference database. For the *C *most frequently occurring acids that we consider, if the frequency of occurrence in the query *Q *is greater than or equal to the frequency of that acid in the reference database scaled by some percentage *E*, then we mark a positive value $v_{f}^{+}$ in a feature vector. Otherwise, we mark a negative value $v_{f}^{-}$, and the result is a *C*-length binary vector. We call this technique the binary frequency transformation, and denote it by $\phi_{f}^{C,E}$(*Q*). We call the combination of the primary structure spectrum kernel and the binary frequency transformation Method PF, and its feature vectors take the form

$$v=<\phi_{s}(\alpha^{k},Q),\phi_{f}^{C,E}(Q)>.$$

For our experiments, we set the following values: *C *= 5, *E *= 90%, $v_{f}^{+}$ = 1, $v_{f}^{-}$ = 0.

Preliminary experiments showed that BLAST and Method P performed approximately equally in the number of types of DNA repair proteins they could outclassify relative to each other. As BLAST considers sequence alignment and Method P considers the global frequency of short polypeptides, it would be useful to combine these two approaches in some way. Additionally, we wish to avoid the high computation time in a homology-generative model such as the Fisher kernel [48]. A previous study reported the use of SVMs as a fallback when homology searching did not yield a positive result in the search for a particular type of protein [13]. We alternatively arrive at a conclusion for a query protein by novelly combining the result of a homology search for a query against a reference database with the primary sequence spectrum kernel. In this technique, it is necessary to have training data and test data that are unbiased, meaning that they can use the same reference database, and this serves as the motivation for introducing the 11R data division method mentioned earlier. Using a BLAST e-value threshold of 0.001, we append to the primary spectrum kernel (Method P) the value $v_{b}^{+}$ if the returned e-value of the query protein is less than the threshold, and otherwise append the value $v_{b}^{-}$ (if no homologous sequence and e-value is returned, +∞ is assigned). Let us call this one dimensional output *φ*_*H*_(*Q*), and hence the combination (which we will call Method PH) of the homology search and spectrum kernel results in feature vectors

**v **= <*φ*_*s *_(*α*^*k*^, *Q*), *φ*_*H*_(*Q*) >.

In practice, we set the values of $v_{b}^{-}$ and $v_{b}^{-}$ to 1 and -1, respectively.

The final SVM-inspired tranformation, named PSH, is intuitively derived from the previous transformations. Method PSH is the combination of a primary sequence spectrum kernel, a secondary structure spectrum kernel, and homology information. By using this transformation, we can maximize the amount of data (features) available to the SVM under our experimental conditions, which can be useful in situations with reduced datasets. Formally, Method PSH has vectors of the form

$$v=<\phi_{s}(\alpha^{k_{1}},Q_{p}),\phi_{s}({\left\{C,E,H\right\}}^{k_{2}},Q_{s}),\phi_{H}(Q)>.$$

These five data transformation techniques are used so that data can be input into Support Vector Machines. Performing DNA repair protein recognition and classification tasks in a generative fashion using BLAST alone is also possible. In this situation, only DNA repair protein training data is used as a BLAST reference database. We do not include the non-repair training data, as we wish for the result of our BLAST to return an e-value that tells us how similar the query protein is to other DNA repair proteins. Testing non-repair query data on a non-repair reference database would not help us assess the ability of BLAST to recognize DNA repair proteins.

## Authors' contributions

JB implemented data division and feature vector programs, authored scripts to perform experiments and collect results, designed and created the INTREPED web service, and drafted the manuscript. TA supervised this work, revised the BLAST-only methodology to include only positive training samples, and provided valuable discussion through its development. Both authors read and approved the manuscript.

## Supplementary Material

Additional File 1 **Additional ROC curves for identification experiments.** ROC curves similar to Figures 1 and 2 for identification experiments performed at 50%, 90%, and unfiltered (0%) datasets. Click here for file

Additional File 2 **Additional classification experiments.** Results for all GO-based repair pathway classification experiments, including pathways not shown in the Results section of this paper. Click here for file

Additional File 3 **Classifier statistical significance tests.** Statistical analysis of multiple classifier performance on identification and classification datasets. Pairwise comparison of classifiers on identification data is also given. Click here for file

Additional File 4 **Detailed multi-genome overlap results.** Per-species tabulation of sequentially "identical" (predicted) DNA repair proteins at 100%, 99%, and 98% similarities using Methods P, PH, and BLAST. Click here for file

Additional File 5 **Repair protein candidates.** The genome name and chromosome location of novel proteins predicted to be related to DNA repair using 31 vertebrate genomes from ENSEMBL. Click here for file

Additional File 6 **Spectrum kernel implementation runtimes.** It is noted in [46] that the trie data structure is an efficient, linear-time implementation of the spectrum kernel. In this file, we briefly discuss how to achieve a 60% reduction in spectrum kernel computation time by using an alternative data structure. This improvement is also utilized by the free and publicly available INTREPED web server. Click here for file

Additional File 7 **Identification and classification datasets.** The PDB and UniProt identification datasets (protein identifiers), as well as the UniProt classification datasets, for each sequence similarity used in this report. Click here for file
