# Supplementary material for: Identification of novel DNA repair proteins via primary sequence, secondary structure, and homology
Source: BMC Bioinformatics. 2009 Jan 20;10:25. doi: 10.1186/1471-2105-10-25 (PMC2660303; doi:10.1186/1471-2105-10-25)
Supplement: Additional File 1 — Additional ROC curves for identification experiments. ROC curves similar to Figures 1 and 2 for identification experiments performed at 50%, 90%, and unfiltered (0%) datasets. [file 1471-2105-10-25-S1.pdf]

Additional file description:

ROC curves of DNA repair protein identification experiments using GO-PDB and GO-UniProt datasets. For data clustered at 90% and 0% similarity, dataset sizes are provided in Tables 2 and 4 of the main manuscript.

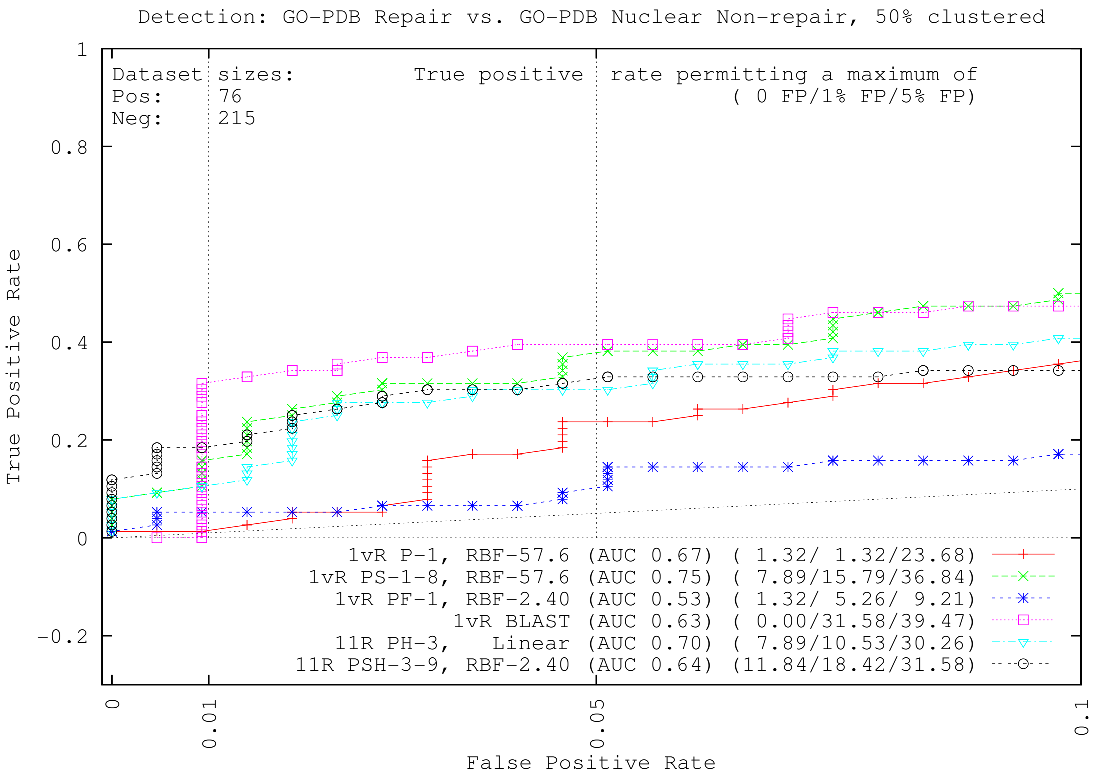

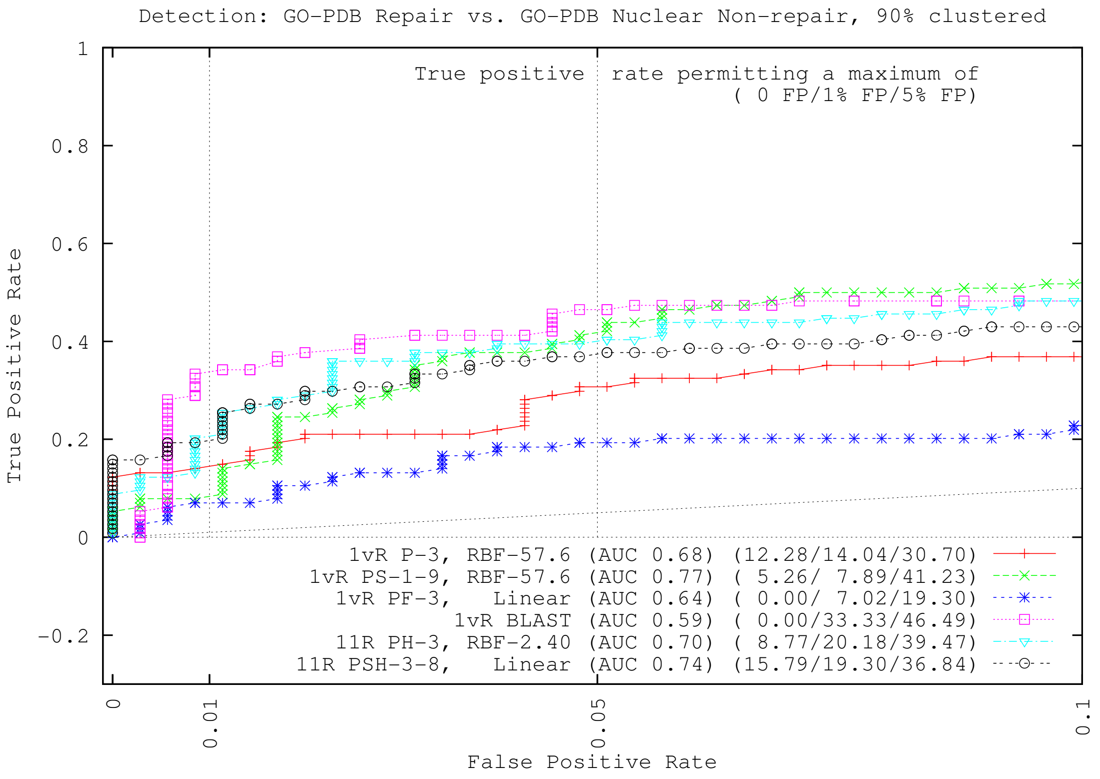

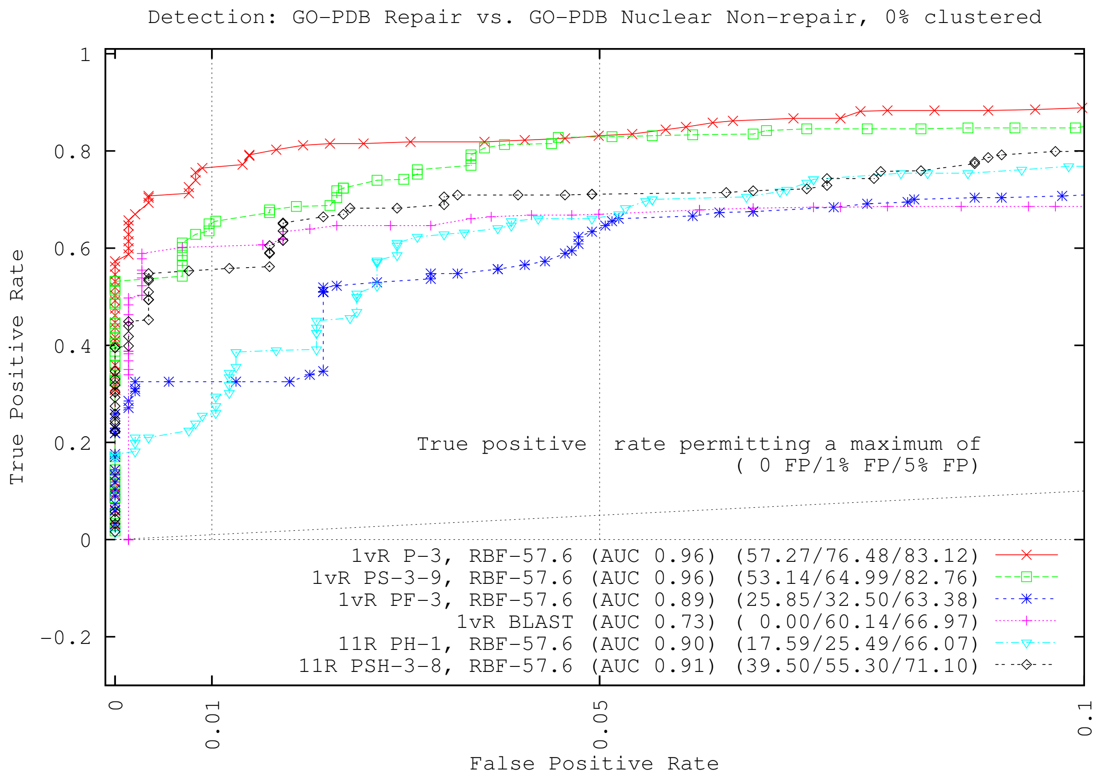
Detection: GO-PDB Repair vs. GO-PDB Nuclear Non-repair, 0% clustered
True Positive Rate
False Positive Rate
True positive rate permitting a maximum of
(0 FP/1% FP/5% FP)
1vR P-3, RBF-57.6 (AUC 0.96) (57.27/76.48/83.12)
1vR PS-3-9, RBF-57.6 (AUC 0.96) (53.14/64.99/82.76)
1vR PF-3, RBF-57.6 (AUC 0.89) (25.85/32.50/63.38)
1vR BLAST (AUC 0.73) (0.00/60.14/66.97)
11R PH-1, RBF-57.6 (AUC 0.90) (17.59/25.49/66.07)
11R PSH-3-8, RBF-57.6 (AUC 0.91) (39.50/55.30/71.10)

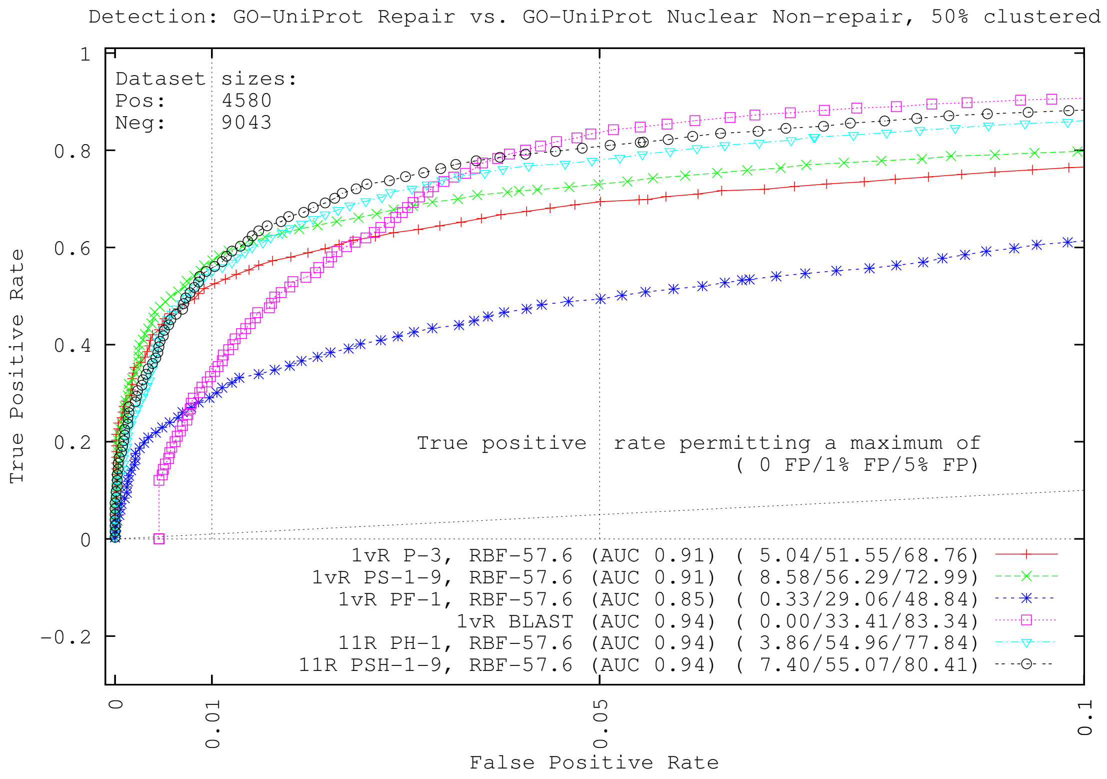
Detection: GO-UniProt Repair vs. GO-UniProt Nuclear Non-repair, 50% clustered
Dataset sizes:
Pos: 4580
Neg: 9043
True Positive Rate
False Positive Rate
True positive rate permitting a maximum of
(0 FP/1% FP/5% FP)
1vR P-3, RBF-57.6 (AUC 0.91) (5.04/51.55/68.76)
1vR PS-1-9, RBF-57.6 (AUC 0.91) (8.58/56.29/72.99)
1vR PF-1, RBF-57.6 (AUC 0.85) (0.33/29.06/48.84)
1vR BLAST (AUC 0.94) (0.00/33.41/83.34)
11R PH-1, RBF-57.6 (AUC 0.94) (3.86/54.96/77.84)
11R PSH-1-9, RBF-57.6 (AUC 0.94) (7.40/55.07/80.41)

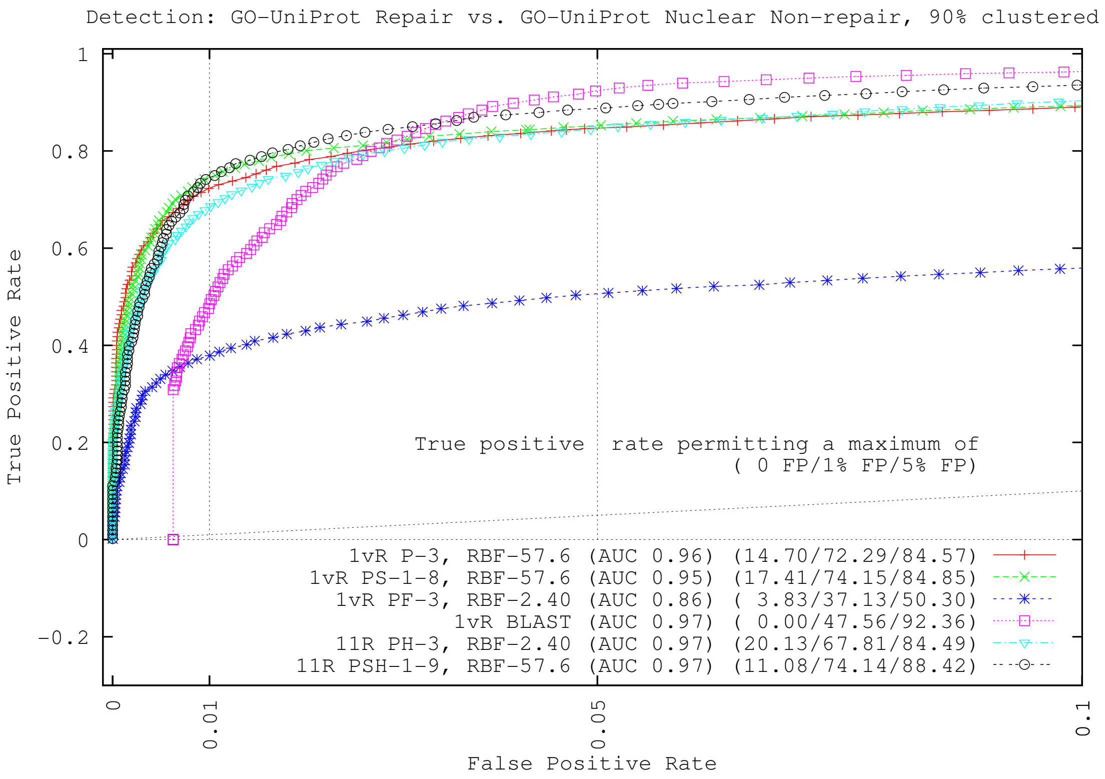
Detection: GO-UniProt Repair vs. GO-UniProt Nuclear Non-repair, 90% clustered
True Positive Rate
False Positive Rate
True positive rate permitting a maximum of
(0 FP/1% FP/5% FP)
1vR P-3, RBF-57.6 (AUC 0.96) (14.70/72.29/84.57)
1vR PS-1-8, RBF-57.6 (AUC 0.95) (17.41/74.15/84.85)
1vR PF-3, RBF-2.40 (AUC 0.86) (3.83/37.13/50.30)
1vR BLAST (AUC 0.97) (0.00/47.56/92.36)
11R PH-3, RBF-2.40 (AUC 0.97) (20.13/67.81/84.49)
11R PSH-1-9, RBF-57.6 (AUC 0.97) (11.08/74.14/88.42)

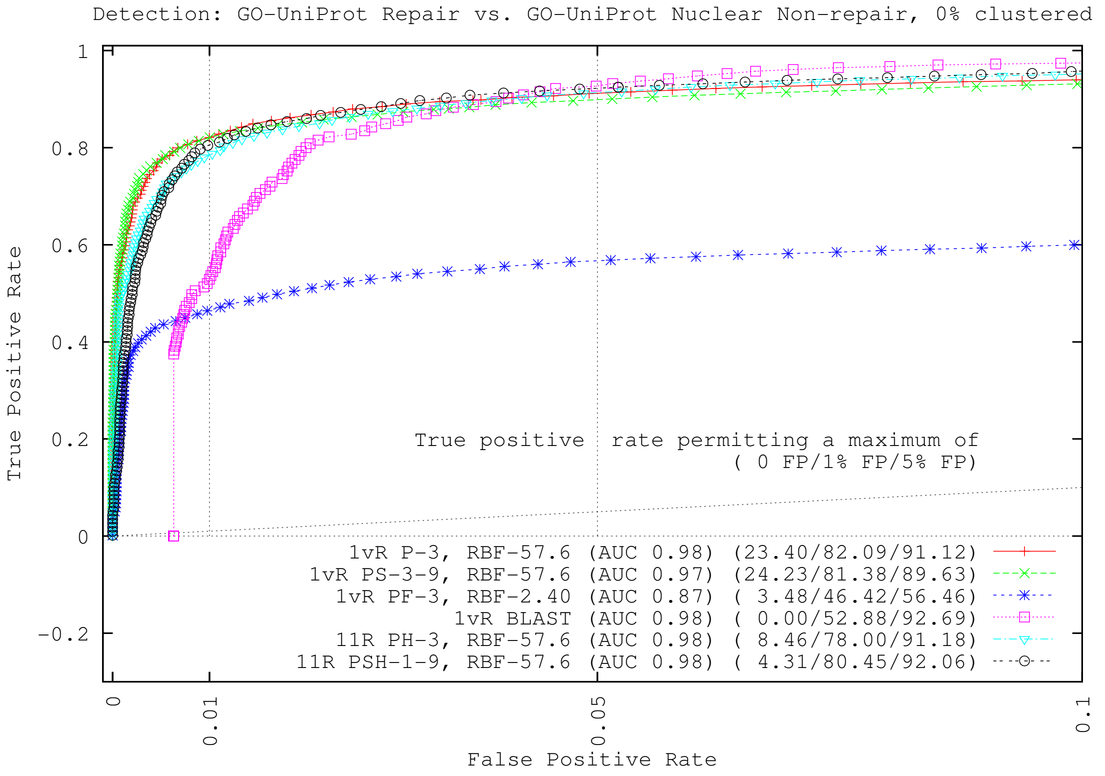
Detection: GO-UniProt Repair vs. GO-UniProt Nuclear Non-repair, 0% clustered
True Positive Rate
False Positive Rate
True positive rate permitting a maximum of
(0 FP/1% FP/5% FP)
1vR P-3, RBF-57.6 (AUC 0.98) (23.40/82.09/91.12)
1vR PS-3-9, RBF-57.6 (AUC 0.97) (24.23/81.38/89.63)
1vR PF-3, RBF-2.40 (AUC 0.87) (3.48/46.42/56.46)
1vR BLAST (AUC 0.98) (0.00/52.88/92.69)
11R PH-3, RBF-57.6 (AUC 0.98) (8.46/78.00/91.18)
11R PSH-1-9, RBF-57.6 (AUC 0.98) (4.31/80.45/92.06)
