# Supplementary material for: Identification of novel DNA repair proteins via primary sequence, secondary structure, and homology
Source: BMC Bioinformatics. 2009 Jan 20;10:25. doi: 10.1186/1471-2105-10-25 (PMC2660303; doi:10.1186/1471-2105-10-25)
Supplement: Additional File 2 — Additional classification experiments. Results for all GO-based repair pathway classification experiments, including pathways not shown in the Results section of this paper. [file 1471-2105-10-25-S2.pdf]

Table 1 - Classification Results: Base Excision Repair

Classification experiments using Base Excision Repair (Gene Ontology ID 0006284) as the type of protein to identify. "Pos/Neg" shows the number of BER-related and non-related proteins for a particular sequence similarity. AUC is the total area under the ROC curve obtained for the experiment. TPR-0, TPR-1%, and TPR-5% indicate the portion (percentage) of true positives identified allowing maximums of 0 false positives, 1% false positives, and 5% false positives, respectively. For each sequence similarity dataset, the top performing method for each metric is underlined (unless a tie exists in more than half of the classifiers). Methods P, PS, PF, and BLAST are performed using 1vR cross-validation, and Methods PH and PSH are performed using 11R (one-versus-one-versus-rest) cross-validation.

Seq. Similarity	Pos/Neg (% Pos)	Method	AUC	TPR-0	TPR-1%	TPR-5%
50%	630/1200 (34%)	P	0.97	11.59	71.75	88.89
		PS	0.97	14.92	73.02	88.73
		PF	0.84	16.03	43.02	45.40
		BLAST	0.96	0.00	83.65	93.65
		PH	0.95	19.37	47.46	80.48
		PSH	0.95	7.14	45.24	86.67
90%	1721/2924 (37%)	P	0.99	56.67	90.53	96.40
		PS	0.99	15.75	91.52	95.35
		PF	0.86	39.80	50.78	53.81
		BLAST	0.97	1.05	91.46	95.35
		PH	0.98	18.94	73.68	94.25
		PSH	0.98	11.56	70.66	95.64
0%	2624/4723 (35%)	P	0.99	41.27	92.57	97.14
		PS	0.99	31.33	91.54	95.96
		PF	0.91	22.48	50.80	62.46
		BLAST	0.98	0.00	56.78	98.17
		PH	0.98	7.01	53.85	90.97
		PSH	0.99	15.85	84.83	96.88

Table 2 - Classification Results: DNA Dealyklation

Classification experiments using DNA Dealyklation (GO:0006307) as the type of protein to identify. "Pos/Neg" shows the number of dealyklation-related and non-related proteins for a particular sequence similarity. Column meanings are identical to Table 1. Rows marked as "N/A" mean that data was insufficient to run experiments (minimum 25 proteins).

Seq. Similarity	Pos/Neg (% Pos)	Method	AUC	TPR-0	TPR-1%	TPR-5%
50% 90%	N/A					
0%	25/7322 (<1%)	P	0.94	60.00	84.00	88.00
		PS	0.96	40.00	84.00	84.00
		PF	0.88	40.00	84.00	84.00
		BLAST	0.94	0.00	72.00	92.00
		PH	0.95	0.00	32.00	88.00
		PSH	0.98	12.00	60.00	92.00

Table 3 - Classification Results: DNA synthesis during DNA Repair

Classification experiments using DNA synthesis during DNA repair (GO:0000731) as the type of protein to identify. "Pos/Neg" shows the number of synthesis-related and non-related proteins for a particular sequence similarity. Column meanings are identical to Table 1. Rows marked as "N/A" mean that data was insufficient to run experiments (minimum 25 proteins).

Seq. Similarity	Pos/Neg (% Pos)	Method	AUC	TPR-0	TPR-1%	TPR-5%
50%	N/A					
90%	N/A					
0%	28/7319 (<1%)	P	0.91	0.00	46.43	67.86
		PS	0.91	0.00	42.86	60.71
		PF	0.79	0.00	28.57	42.86
		BLAST	0.91	0.00	<u>82.14</u>	<u>82.14</u>
		PH	<u>0.94</u>	0.00	46.43	78.57
		PSH	0.91	0.00	60.71	71.43

Table 4 - Classification Results: Double Strand Break Repair

Classification experiments using Double Strand Break repair (GO:0006302) as the type of protein to identify. "Pos/Neg" shows the number of DSB repair-related and non-related proteins for a particular sequence similarity. Column meanings are identical to Table 1.

Seq. Similarity	Pos/Neg (% Pos)	Method	AUC	TPR-0	TPR-1%	TPR-5%
50%	174/1656 (10%)	P	0.82	9.77	24.71	40.23
		PS	<u>0.89</u>	<u>25.86</u>	54.02	66.67
		PF	0.80	9.77	17.82	35.63
		BLAST	0.88	15.52	<u>64.37</u>	<u>73.56</u>
		PH	0.83	5.75	17.82	47.70
		PSH	<u>0.89</u>	1.72	25.29	64.94
90%	266/4379 (6%)	P	0.92	16.92	55.64	71.05
		PS	<u>0.94</u>	<u>27.44</u>	69.92	77.82
		PF	0.90	<u>27.44</u>	46.62	67.29
		BLAST	0.92	13.53	<u>76.69</u>	<u>83.08</u>
		PH	0.89	0.38	19.92	40.23
		PSH	0.93	7.14	34.59	67.67
0%	364/6983 (5%)	P	<u>0.94</u>	26.65	63.19	75.82
		PS	<u>0.94</u>	<u>34.62</u>	73.90	83.24
		PF	0.91	26.65	58.24	69.51
		BLAST	0.93	0.00	<u>78.30</u>	<u>86.81</u>
		PH	0.87	2.47	10.71	29.12
		PSH	0.93	2.75	29.12	60.71

Table 5 - Classification Results: Error-prone DNA Repair

Classification experiments using Error-prone DNA repair (GO:0045020) as the type of protein to identify. "Pos/Neg" shows the number of error-prone repair-related and non-related proteins for a particular sequence similarity. Column meanings are identical to Table 1. Rows marked as "N/A" mean that data was insufficient to run experiments (minimum 25 proteins).

Seq. Similarity	Pos/Neg (% Pos)	Method	AUC	TPR-0	TPR-1%	TPR-5%
50%	N/A					
90%	36/4609 (<1%)	P	1.00	100.00	100.00	100.00
		PS	1.00	100.00	100.00	100.00
		PF	0.99	52.78	97.22	100.00
		BLAST	0.99	0.00	100.00	100.00
		PH	0.99	52.78	100.00	100.00
		PSH	1.00	100.00	100.00	100.00
0%	46/7301 (<1%)	P	0.99	97.83	100.00	100.00
		PS	0.99	93.48	100.00	100.00
		PF	0.99	84.78	100.00	100.00
		BLAST	0.99	0.00	100.00	100.00
		PH	0.99	84.78	100.00	100.00
		PSH	0.99	84.78	100.00	100.00

Table 6 - Classification Results: Mismatch Repair

Classification experiments using Mismatch repair (GO:0006289) as the type of protein to identify. "Pos/Neg" shows the number of mismatch repair-related and non-related proteins for a particular sequence similarity. Column meanings are identical to Table 1.

Seq. Similarity	Pos/Neg (% Pos)	Method	AUC	TPR-0	TPR-1%	TPR-5%
50%	468/1362 (26%)	P	0.97	41.03	79.06	89.53
		PS	0.94	43.80	67.95	79.06
		PF	0.95	15.60	57.48	79.06
		BLAST	0.98	47.86	97.01	97.22
		PH	0.97	11.11	57.69	94.02
		PSH	0.98	9.62	72.86	96.58
90%	1020/3625 (22%)	P	0.99	58.33	91.76	96.96
		PS	0.99	70.69	87.06	93.14
		PF	0.97	37.06	72.35	88.92
		BLAST	0.99	0.00	99.31	99.31
		PH	0.97	15.98	39.51	90.10
		PSH	0.98	12.45	55.29	94.22
0%	1777/5570 (24%)	P	0.99	59.31	92.97	96.85
		PS	0.99	59.31	92.18	95.10
		PF	0.96	31.57	74.23	86.10
		BLAST	0.98	0.00	93.47	98.59
		PH	0.97	16.83	56.27	86.21
		PSH	0.98	16.88	71.64	87.73

Table 7 - Classification Results: Nucleotide Excision Repair

Classification experiments using Nucleotide Excision Repair (GO:0006289) as the type of protein to identify. "Pos/Neg" shows the number of NER-related and non-related proteins for a particular sequence similarity. Column meanings are identical to Table 1.

Seq. Similarity	Pos/Neg (% Pos)	Method	AUC	TPR-0	TPR-1%	TPR-5%
50%	363/1467 (20%)	P	0.90	37.47	50.69	66.12
		PS	0.91	35.54	56.75	71.07
		PF	0.86	22.04	41.60	56.47
		BLAST	0.95	53.99	84.85	88.43
		PH	0.94	2.75	43.25	79.89
		PSH	0.95	4.68	56.75	87.88
90%	1325/3320 (29%)	P	0.98	81.58	87.55	92.23
		PS	0.98	80.23	88.83	92.45
		PF	0.88	25.89	39.77	63.77
		BLAST	0.98	0.00	94.19	97.13
		PH	0.98	4.15	75.02	95.25
		PSH	0.98	1.43	84.53	96.45
0%	2106/5241 (29%)	P	0.98	37.65	89.27	94.16
		PS	0.98	65.19	90.41	93.16
		PF	0.88	21.13	39.55	54.37
		BLAST	0.97	0.00	90.03	96.96
		PH	0.97	3.23	64.15	82.15
		PSH	0.98	11.49	79.49	91.36

Table 8 - Classification Results: Postreplication Repair

Classification experiments using Postreplication repair (GO:0006301) as the type of protein to identify. "Pos/Neg" shows the number of postreplication-related and non-related proteins for a particular sequence similarity. Column meanings are identical to Table 1. Rows marked as "N/A" mean that data was insufficient to run experiments (minimum 25 proteins).

Seq. Similarity	Pos/Neg (% Pos)	Method	AUC	TPR-0	TPR-1%	TPR-5%
50% 90%	N/A					
0%	28/7319 (<1%)	P	0.87	17.86	32.14	57.14
		PS	0.89	14.29	46.43	64.29
		PF	0.81	0.00	32.14	53.57
		BLAST	0.72	0.00	14.29	28.57
		PH	0.89	7.14	39.29	64.29
		PSH	0.86	0.00	46.43	60.71

Table 9 - Classification Results: Regulation of DNA Repair

Classification experiments using "Regulation of DNA repair" (GO:0006282) as the type of protein to identify. "Pos/Neg" shows the number of regulation-related and non-related proteins for a particular sequence similarity. Column meanings are identical to Table 1.

Seq. Similarity	Pos/Neg (% Pos)	Method	AUC	TPR-0	TPR-1%	TPR-5%
50%	114/1716 (6%)	P	0.97	57.02	92.98	94.74
		PS	0.98	87.72	93.86	95.61
		PF	0.96	51.75	92.11	93.86
		BLAST	0.96	85.96	94.74	94.74
		PH	0.99	50.88	93.86	97.37
		PSH	0.99	76.32	93.86	97.37
90%	174/4471 (4%)	P	0.98	62.64	94.83	97.13
		PS	0.99	90.80	95.40	97.70
		PF	0.97	52.30	93.68	94.83
		BLAST	0.99	82.76	97.13	97.13
		PH	0.99	62.07	97.13	98.28
		PSH	0.99	83.33	95.98	97.70
0%	264/7083 (4%)	P	0.99	69.70	97.35	98.48
		PS	0.99	88.26	96.97	99.24
		PF	0.99	36.74	95.83	97.35
		BLAST	0.99	21.97	98.48	98.48
		PH	0.99	36.74	98.86	99.24
		PSH	0.99	80.68	98.11	99.24

Table 10 - Classification Results: Single Stand Break Repair

Classification experiments using Single Strand Break repair (GO:0006282) as the type of protein to identify. "Pos/Neg" shows the number of SSB-related and non-related proteins for a particular sequence similarity. Column meanings are identical to Table 1. Rows marked as "N/A" mean that data was insufficient to run experiments (minimum 25 proteins).

Seq. Similarity	Pos/Neg (% Pos)	Method	AUC	TPR-0	TPR-1%	TPR-5%
50%		N/A				
90%	25/1805 (1%)	P	0.99	76.00	96.00	100.00
		PS	0.96	4.00	84.00	96.00
		PF	0.99	4.00	80.00	96.00
		BLAST	0.99	0.00	100.00	100.00
		PH	0.99	4.00	100.00	100.00
		PSH	0.99	8.00	96.00	100.00
0%	40/4605 (1%)	P	0.99	30.00	97.50	100.00
		PS	0.97	25.00	95.00	97.50
		PF	0.95	25.00	87.50	92.50
		BLAST	0.99	0.00	100.00	100.00
		PH	0.99	0.00	100.00	100.00
		PSH	0.99	22.50	100.00	100.00
