# Supplementary material for: Identification of novel DNA repair proteins via primary sequence, secondary structure, and homology
Source: BMC Bioinformatics. 2009 Jan 20;10:25. doi: 10.1186/1471-2105-10-25 (PMC2660303; doi:10.1186/1471-2105-10-25)
Supplement: Additional File 3 — Classifier statistical significance tests. Statistical analysis of multiple classifier performance on identification and classification datasets. Pairwise comparison of classifiers on identification data is also given. [file 1471-2105-10-25-S3.pdf]

Table 1 - Multi-classifier statistical analysis

Using the Kruskal-Wallis test on each of the identification and classification datasets at 50%, 90%, and 0% (unfiltered) similarity, the statistical likelihood that all of the methods presented in this paper produce the same average performance using 5-fold cross validation is listed in Table 1. For each dataset and sequence similarity pair, we select the AUC and either the TPR-1% or TPR-5% metric as our performance values to test for statistical significance. When the AUC-based probability is less than 0.05 (highlighted in bold), we reject the null hypothesis that the methods are equivalent. Results for repair protein types with insufficient data at or below 90% sequence similarity are not shown.

		Sequence similarity		
Dataset	Statistic	50%	90%	0%
GO-PDB	AUC	0.2448	0.1167	**0.0191**
	TPR-5%	0.9102	0.6320	0.2962
GO-UniProt	AUC	0.2502	0.2338	0.8775
	TPR-1%	0.0186	0.0084	0.01791

Above: Identification; Below: Classification

BER	AUC	0.5135	0.9368	0.3394
	TPR-5%	0.2909	0.9920	0.9072
DSB Repair	AUC	0.1081	0.9565	0.9600
	TPR-5%	0.2011	0.4591	0.8005
Mismatch Repair	AUC	0.2079	0.5053	0.7287
	TPR-5%	0.7022	0.1415	0.4163
NER	AUC	0.0851	0.4396	0.2238
	TPR-5%	0.0079	0.3696	0.5692
Regulation of DNA repair	AUC	0.7976	0.7233	0.3092
	TPR-5%	0.9430	0.9200	0.6982
SSB Repair	AUC	-	0.1450	0.4896
	TPR-5%	-	0.4060	0.4060

Table 2 - Pairwise comparison of classifiers on GO-PDB data.

Using both the (parametric) t-test (T) and the (non-parametric) Wilcoxon Signed-Rank Test (W) on the GO-PDB datasets filtered at 50%, 90%, and unfiltered similarity, the probability that each pair of classifiers produces the same AUC score on average is listed. Bold values indicate the statistical difference is significant at the 0.05 confidence level and we can reject the null hypothesis that the classifiers are equivalent on average. The table for 0% sequence similarity is the same table given in the main text as Table 3.

		50% sequence similarity			
Methodology	Test	P	PS	PH	PSH
PS	T	0.4028			
	W	0.4206			
PH	T	0.7314	0.4064		
	W	0.8413	0.5476		
PSH	T	0.7352	0.2065	0.4423	
	W	0.8413	0.2222	0.4206	
BLAST	T	0.4687	**0.0109**	0.1111	0.7350
	W	0.4206	**0.0159**	0.1508	1.0000

		90% sequence similarity			
	Test	P	PS	PH	PSH
PS	T	0.1438			
	W	0.2222			
PH	T	0.8362	0.3109		
	W	0.8413	0.5476		
PSH	T	0.2947	0.6214	0.5022	
	W	0.5476	0.9166	0.5476	
BLAST	T	0.1291	0.1395	0.1296	0.2520
	W	0.2222	**0.0159**	0.1508	0.0556

		0% sequence similarity			
	Test	P	PS	PH	PSH
PS	T	0.7153			
	W	0.8413			
PH	T	0.0844	0.1287		
	W	0.0952	0.1508		
PSH	T	0.1006	0.1705	0.7109	
	W	0.1508	0.2222	0.8413	
BLAST	T	**0.0094**	**0.0104**	0.2442	**0.0193**
	W	**0.0159**	**0.0159**	0.0952	0.0556

Table 3 - Pairwise comparison of classifiers on GO-UniProt data.

Using both the (parametric) t-test (T) and the (non-parametric) Wilcoxon Signed-Rank Test (W) on the GO-UniProt datasets filtered at 50%, 90%, and unfiltered similarity, the probability that each pair of classifiers produces the same AUC score on average is listed. Here, the statistical results suggest that while classifiers will vary to a small degree in their prediction performance (AUC), the large UniProt datasets provide enough training examples that the various classification models produce similar results. No classifier produces average results identical to another classifier with a probability of less than 0.05, using either statistical test.

Methodology	Test	P	PS	PH	PSH
	50% sequence similarity				
PS	T	0.9160			
	W	0.6905			
PH	T	0.2643	0.3973		
	W	0.2222	0.2222		
PSH	T	0.1497	0.2600	0.6944	
	W	0.2222	0.2222	0.4206	
BLAST	T	0.1046	0.2181	0.6027	0.9655
	W	0.0952	0.3095	1.0000	0.5476

	90% sequence similarity				
	Test	P	PS	PH	PSH
PS	T	0.8028			
	W	0.6905			
PH	T	0.4027	0.3169		
	W	0.3095	0.2222		
PSH	T	0.2182	0.1782	0.7028	
	W	0.3095	0.2222	0.8413	
BLAST	T	0.1317	0.1225	0.5574	0.8968
	W	0.0952	0.0556	0.8413	0.8413

	0% sequence similarity				
	Test	P	PS	PH	PSH
PS	T	0.6888			
	W	0.6905			
PH	T	0.6050	0.4040		
	W	0.6905	0.4206		
PSH	T	0.6030	0.4018	0.9968	
	W	0.6905	0.5476	1.0000	
BLAST	T	0.7525	0.4772	0.6951	0.6909
	W	0.6905	0.6905	0.6905	0.6905
