# Supplementary material for: Identification of novel DNA repair proteins via primary sequence, secondary structure, and homology
Source: BMC Bioinformatics. 2009 Jan 20;10:25. doi: 10.1186/1471-2105-10-25 (PMC2660303; doi:10.1186/1471-2105-10-25)
Supplement: Additional File 6 — Spectrum kernel implementation runtimes. It is noted in [46] that the trie data structure is an efficient, linear-time implementation of the spectrum kernel. In this file, we briefly discuss how to achieve a 60% reduction in spectrum kernel computation time by using an alternative data structure. This improvement is also utilized by the free and publicly available INTREPED web server. [file 1471-2105-10-25-S6.pdf]

# Additional file

It is noted in [1] that the trie data structure is an efficient, linear-time implementation of the spectrum kernel. As an alternative, we consider the following approach.

For each symbol in our set of accepted input symbols $I$ (e.g., $\alpha$ as in the Problem Definition or $\{C, E, H\}$ for secondary structure), we assign a bijection $B : I \rightarrow Z^+$ so that each symbol has a unique value between 1 and $|I|$. Then, for each $k$-mer $s = s_1 s_2 \ldots s_k$ that we want to count or we encounter in an amino acid sequence, we can calculate a unique index

$$index(s) = |I|^{k-1}B(s_1) + |I|^{k-2}B(s_2) + \ldots + |I|B(s_{k-1}) + B(s_k).$$

This calculation (a perfect hash requiring an array of $|I|^k$ integers) can be done in $O(n)$ time in the length of the sequence, similar to the trie data structure. However, the constant time factors of the trie node creation and retrieval are larger than the constant time factors in our implementation. The included figure shows the computation times for both trie-based and hash-based spectrum kernel implementations, as well as a comparison with an implementation using the C++ Standard Template Library [2] map structure. As can be seen from the figure, we achieve computational time reduction of approximately 60% relative to the map and often-used trie methods.

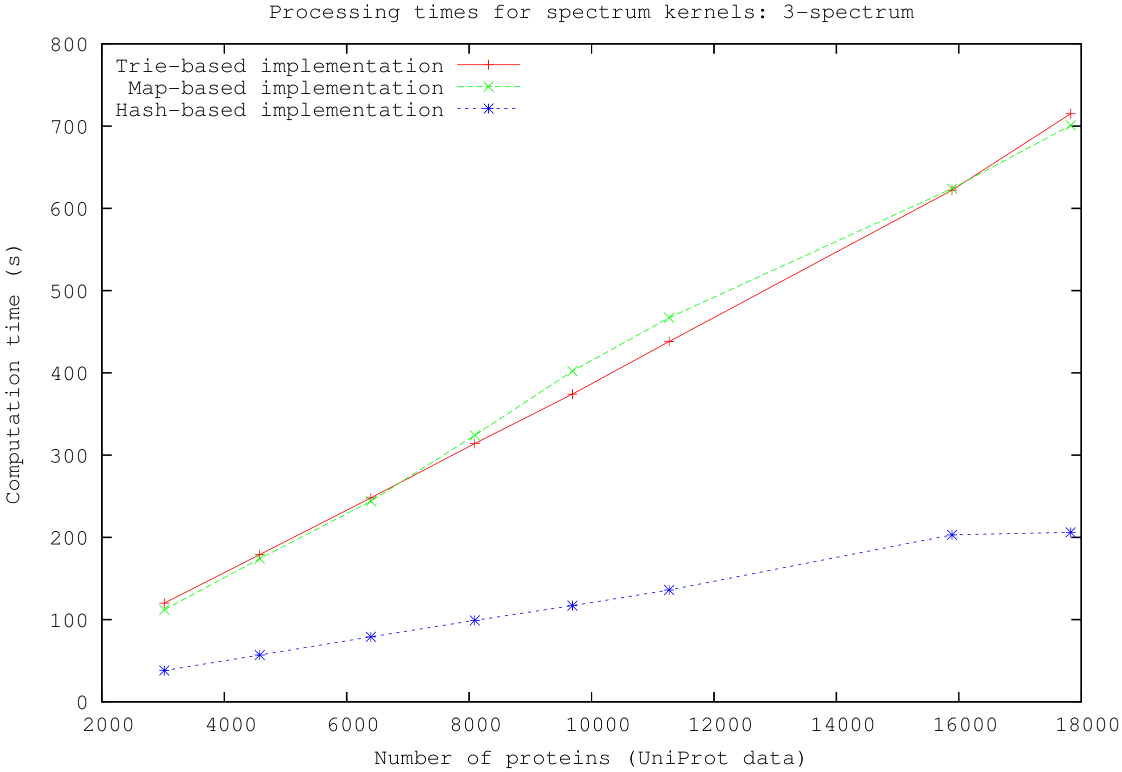


# References


[1] Leslie C, Eskin E, Noble WS: **The spectrum kernel: A string kernel for SVM protein classification.** In *Proceedings of the Pacific Symposium on Biocomputing: 3-7 January 2002; Lihue.* 2002:566-575.

[2] Stroustrup B: *The C++ Programming Language, Third Edition* Reading: Addison-Wesley Publishing Company; 1998.
